# Plants and traditional knowledge: An ethnobotanical investigation on Monte Ortobene (Nuoro, Sardinia)

**DOI:** 10.1186/1746-4269-5-6

**Published:** 2009-02-10

**Authors:** Maria Adele Signorini, Maddalena Piredda, Piero Bruschi

**Affiliations:** 1 Dipartimento di Biologia Vegetale dell'Università, piazzale delle Cascine 28, I-50144 Firenze, Italy; 2 Vicolo G. Giusti 4, 08100 Nuoro, Italy

## Abstract

**Background:**

Most of the traditional knowledge about plants and their uses is fast disappearing as a consequence of socio-economic and land use changes. This trend is also occurring in areas that are historically exposed to very few external influences, such as Sardinia (Italy). From 2004 to 2005, an ethnobotanical investigation was carried out in the area of Monte Ortobene, a mountain located near Nuoro, in central Sardinia.

**Methods:**

Data were collected by means of semi-structured interviews. All the records – defined as 'citations', i.e. a single use reported for a single botanical species by a single informant – were filed in a data base ('analytical table'), together with additional information: i.e. local names of plants, parts used, local frequencies, and habitats of plants, etc. In processing the data, plants and uses were grouped into general ('categories') and detailed ('secondary categories') typologies of use. Some synthetic indexes have also been used, such as Relative Frequency of Citation (RFC), Cultural Importance Index (CI), the Shannon-Wiener Index (H'), and Evenness Index (J).

**Results:**

Seventy-two plants were cited by the informants as being traditionally used in the area. These 72 'ethnospecies' correspond to 99 botanical taxa (species or subspecies) belonging to 34 families. Three-hundred and one citations, 50 secondary categories of use, and 191 different uses were recorded, most of them concerning alimentary and medicinal plants.

For the alimentary plants, 126 citations, 44 species, and 13 different uses were recorded, while for the medicinal plants, there were 106 citations, 40 species, and 12 uses. Few plants and uses were recorded for the remaining categories. Plants and uses for each category of use are discussed. Analyses of results include the relative abundance of botanical families, wild vs. cultivated species, habitats, frequency, parts of plant used, types of use, knowledge distribution, and the different cultural importance of the species in question.

**Conclusion:**

The study provides examples of several interesting uses of plants in the community, which would seem to show that the custom of using wild plants is still alive in the Monte Ortobene area. However, many practices are no longer in use, and survive only as memories from the past in the minds of elderly people, and often only in one or just a few informants. This rapidly vanishing cultural diversity needs to be studied and documented before it disappears definitively.

## Background

Documenting and safeguarding Traditional Ecological Knowledge (TEK) have become central issues in the planning and management of natural resources. Traditional knowledge of plants and their uses is the result of thousands of years of experience (both apprenticeship and self-directed learning-while-doing) and educational methods (e.g. telling stories). The relevance of this knowledge as far as increasing the daily living standards of rural populations is concerned, as well as in taking decisions regarding the sustainable use of plant resources, has frequently been noted ([1,2]).

However, in developed countries, this traditional knowledge is being widely threatened by current trends of economic globalization that promote intensive agriculture, industrialization, and the migration of rural populations to urban areas (on this subject, see among others, [3]). Consequently, it is crucial to record this fast-disappearing knowledge before it vanishes definitively along with the present generation of elderly persons.

Due to its geographic isolation, the island of Sardinia (Italy) has been exposed to very few external influences, and up until now this has promoted a very high cultural homogeneity and stability. In Sardinia, people are still very proud of their traditional cultural heritage, and a lot of "folk" uses of plants are still maintained, especially in rural and mountain areas. On this subject, see, among others, [4-15].

The aim of this study is to record local knowledge regarding plants and their traditional uses and to assess how this knowledge is distributed within a community of central Sardinia (Italy) situated in the area of Barbagia, one of the richest areas as far as ancient popular traditions are concerned.

The study area, which is located to the west of the town of Nuoro (40°19'16" N, 3°04'05" W) in central Sardinia, is approximately 40 sq km wide. Monte Ortobene is a mountain whose altitudes range up to 995 m. Due to its proximity to the city (approximately 8 km) and easy access, it is one of the areas most frequented by the local community. Only a few natives still live on Monte Ortobene, while many people come here from the nearby city of Nuoro for the purposes of recreational hiking. The economy of the Nuoro municipality, which is the largest populated center in Barbagia, is based mainly on agriculture, handicrafts, and tourism. The population of the entire municipality reaches approximately 36,000 inhabitants (164,000 in the province of Nuoro), with a density of 190.73 inhabitants per sq km. The climate is typically Mediterranean, with rainfall concentrated during the autumn and winter and hot, very dry summers. The prevailing geological substrate is granite. The local vegetation consists mainly of different stages of Mediterranean series (maquis, scrubs, grasslands). The first definite evidence of human settlement in this area dates from 3800 B.C. to 2800 B.C, as attested by the presence of collective hypogeous graves called *Domus de Janas *('Fairy houses').

## Methods

The investigation was carried out from 2004 to 2005. Information was collected on traditional uses of wild plants and also of cultivated ones; however, in the latter case, only uses different from those for which each plant is commonly grown were considered (e. g. using wheat to decorate altars). Uses concerning materials derived from plants but thoroughly processed (e.g. vinegar, bread) were not recorded.

Special care was taken in choosing the informants. Only people who were born and had always lived in the area were taken into consideration, and we also made sure that the source of their knowledge about local uses of plants had only been a question of traditional culture. Due to these strict criteria, only 17 informants were selected and involved in the study. Information was collected by means of semi-structured interviews, because when the informants were interviewed, the detailed prearranged questionnaires proved to be too rigid. The interviews were characterized by empathy and confidence with the informants, together with an attitude of listening and respect, on the part of the interviewer. See [16] for a discussion on the selecting of informants and the conducting of interviews in ethnobotanical investigations. Interviews were carried out in the local language (*limba sarda*) and in Italian.

Voucher specimens of the cited plant species were prepared and deposited in the FIAF (University of Florence, Erbario dei laboratori di Botanica Agraria e forestale) herbarium. Systematic arrangement and nomenclature mostly followed Pignatti's Flora d'Italia [17]. In a few instances of plants belonging to critical groups (e.g.: *Taraxacum officinale *Weber), we resorted to a broad concept of the species (species *sensu lato*), as this appeared to be more suitable when dealing with ethnobotanical data.

All the collected data were filed in a data-base (*analytical table*) consisting of a spreadsheet (Windows Excel 2003). The rows on it corresponded to the species reported, and the columns contained information on each species. As discussed in a previous contribution ([18]), each row in the analytical table represents an elementary record and is intended as a *citation*, i.e. a single use reported for a single species by a single informant. Each citation is supplemented by the following data (see below for explanations):

Scientific name (species or subspecies)

Botanical family

Local vernacular name(s)

Part(s) of the plant used

Category of use (i.e. general typology)

Secondary category of use (i.e. detailed typology)

Way of use

Whether the plant is wild or cultivated

Local frequency

Habitat

Notes

For medicinal plants, further information was also recorded (see below).

Apart from scientific names and classification, all the data were reported just as they had been related by the informants.

*Category of use *is defined as one of the following general typologies of use (for categories marked with an *, no use was reported in this investigation):

Agropastoral

Alimentary

Domestic

Drugs and cigarettes*

Handicrafts

Hunting and fishing*

Ludic

Magical/medicinal

Magical/Ritual/Propitiatory

Medicinal

Religious

Veterinary*

Note that many uses pertaining to all categories included some magical aspect, but only those in which a magical element was predominant were included in the 'Magical/Ritual/Propitiatory' category. Furthermore, uses were included in the handicrafts category only if the material involved was processed or converted in appreciable ways and the product is durable.

*Secondary category of use *is intended as a detailed typology identified within each category. In filing and processing data, secondary category of use was assumed to be the most detailed level in discriminating different uses from one another. For medicinal plants, secondary categories coincided with general therapeutic indications, such as dermatological affections, diseases of the digestive system, etc. Moreover, since uses in this category are more complex and articulate, additional information has been provided: detailed therapeutic indications (e.g. gastritis, nausea, etc.), way of preparation, and way of administration. Olive oil was taken into consideration as a phytoterapeutic remedy only when it was considered by the informant to be an effective component of the medicament, and not merely a solvent or medium, as was the case for ointments or macerations in oil.

*Way of use *is a short description of how the plant is used: preparation, recipes, etc.

In *Notes*, possible further information is reported, such as proverbs, toponyms, notes on past frequency, etc.

We considered as distinct citations those differing from one another in at least one of the following data: species, informant, secondary category of use. Citations differing in minor aspects, such as the part of the plant used, the way of use, and (in medicinal uses) detailed therapeutic indication, way of preparation, and the way of administration were combined into a single citation. Each citation coincides with a single row in the analytical table.

The number of *uses *was obtained by considering as distinct uses for each species those differing in secondary category. Therefore, citations reported for the same secondary category by different informants were counted only once.

We applied quantitative analysis of the data through the use of certain synthetic indexes. Despite the fact that we collected information from only a small number of informants, we tested this quantitative approach in order to verify whether it could improve understanding and interpretation of the results of our investigation. We intended to assess whether synthetic indexes could help to quantify the distribution and diversity of information in each species of plant and/or in each informant, and also the different relevance of each botanical species, even while dealing with very few data. The following synthetic indexes were used:

*Local importance *of each species was calculated by using the Relative Frequency of Citation (RFC) [19]:

$$\text{RFC}=\frac{\text{FC}}{\text{N}}$$

where FC is the number of informants who mentioned the use of the species and N is the total number of informants (17 in this study).

*Diversity of information *within each species was calculated using two different indexes:

- the Cultural Importance Index (CI_s_), calculated by using the formula suggested in [19]:

$${\text{CI}}_{\text{S}}=\sum_{\text{u}={\text{u}}_{1}}^{{\text{u}}_{\text{NC}}}\sum_{\text{i}={\text{i}}_{1}}^{{\text{i}}_{\text{N}}}{\text{UR}}_{\text{ui}}/\text{N}$$

where u is the category of use, NC is the total number of different categories of use (of each 'i' species), UR is the total number of use-reports for each species (corresponding in the present study to 'citations' as defined above), and N is the total number of informants (17 in this study). In calculating NC, defined in [19] as the 'total number of use-categories', for each species we considered the number of different 'secondary categories of use' as defined above. However, it must be noted that these were much more detailed and numerous than the 'use-categories' adopted in [19].

- the Shannon-Wiener Index (H'), calculated by using the formula:

$$\text{H}’=-\sum_{\text{i}=1}^{\text{u}}\left(\text{pi})(\log\text{pi}\right)\text{ base 1}0$$

where u is the number of uses and

$$\text{pi}={\text{n}}_{\text{i}}/\text{N},\text{N}=\sum_{\text{i}=1}^{\text{u}}\text{n}$$

where n_i _is the number of citations for each use (as explained previously, a citation is a single use reported for a single species by a single informant) and N is the total number of citations. This index has been widely employed in order to quantify knowledge distribution in different communities [20]. Here, however, it should be noted that it is applied to a diversity of uses, both within each species and between different species.

*Evenness *was calculated with the Evenness Index (J), using the formula:

$$\text{J}=\frac{\text{H'}}{\log_{{\text{UR}}_{\text{ui}}}}$$

where H' is the Shannon-Wiener Index and UR_ui _is the number of uses reported for each species.

## Results and discussion

### Informants

As pointed out above, only 17 informants were interviewed: three men (17.6%) and 14 women (82.4%). This uneven repartition is not unusual in ethnobotanical investigations in Italy (see, for instance, [13,21]), as a consequence of the importance of women in the domestic context, which is where most plant resources, especially alimentary and medicinal plants, are managed. This also means that, in the current investigation, women proved to be the main upholders of traditions linked to domestic life. Only one informant (5.9%) was younger than 60; 10 (58.8%) were 61–75 years old, and 6 (35.3%) were over 65. Most of them (15, i.e. 88.2%) were retired; only two (11.8%) were still working. Women worked mostly as housewives (8, i.e. 47.1% of all the informants). As for educational qualifications, 9 (52.9%) had had only a primary-school ('scuola elementare') education; one (5.9%), a grammar-school ('scuola media') education; 6 (35.3%) a high-school ('scuola superiore') education; and only one (5.9%) had a university degree. See Table 1 for summarized information on the informants.

**Table 1:** Informants – Demographic data

	**n**	***%***
**Age**		

Under 60	1	*5.9*

61–75	10	*58.8*

Above 75	6	*35.3*

**Sex**		

Men	3	*17.6*

Women	14	*82.4*

**Education**		

Primary school	9	*52.9*

Secondary school	1	*5.9*

High school	6	*35.3*

University degree	1	*5.9*

**Occupation**		

Housewives	8	*47.1*

Employees	5	*29.4*

Hospital nurses	3	*17.6*

Others	1	*5.9*


Retired	15	*88.2*

Employed	2	*11.8*

### Plants. General considerations

The analytical table from which all other tables and elaborations are derived is not reported here, but is available (in Italian) at .

The main results on informants, plants and uses are reported in several synthetic tables (Tables 1, 2, 3, 4, 5, 6, 7, 8, 9 and 10). For a full list of all the species recorded during this investigation, see Additional file 1.

**Table 2:** Ethnobotanical plants – Synthesis of main results

Number of species	99
Number of citations	301

Number of uses	191

Number of different secondary categories of use	50

**Table 3:** Ethnobotanical plants – Categories of use

**Category**	**Number of citations**	**Number of species**	**Number of uses (secondary categories)**
Alimentary	126	44	13

Medicinal	106	40	12

Domestic	23	15	8

Ludic	21	15	5

Agropastoral	8	7	5

Magic/Medicinal	6	5	2

Religious	7	4	1

Handicraft	2	2	2

Magic/ritual/propitiatory	2	2	2

**Table 4:** Ethnobotanical plants – Botanical families

	**Number of species**	**Number of citations**
Compositae	22	57

Liliaceae	7	36

Rosaceae	7	24

Umbelliferae	6	19

Malvaceae	4	27

Labiatae	4	10

Urticaceae	4	9

Leguminosae	4	8

Graminaceae	4	7

Cruciferae	4	6

Apocynaceae	3	5

Cistaceae	3	3

Oleaceae	2	9

Myrtaceae	2	5

Plantaginaceae	2	4

Boraginaceae	2	4

Crassulaceae	2	4

Chenopodiaceae	1	16

Cactaceae	1	9

Ericaceae	1	7

Lauraceae	1	5

Araliaceae	1	4

Fagaceae	1	3

Guttiferae	1	3

Polygonaceae	1	3

Anacardiaceae	1	3

Papaveraceae	1	2

Juncaceae	1	2

Pinaceae	1	2

Euphorbiaceae	1	1

Ranunculaceae	1	1

Scrophulariaceae	1	1

Caprifoliaceae	1	1

Palmae	1	1

**Table 5:** Ethnobotanical plants – Wild/cultivated

	**Number of species**
Wild	89

Cultivated	6

Wild and cultivated	3

Not present in the area	1

**Table 6:** Ethnobotanical plants – Knowledge distribution

	**Number of species**
Mentioned by 12 informants	1

Mentioned by 11 informants	0

Mentioned by 10 informants	0

Mentioned by 9 informants	1

Mentioned by 8 informants	0

Mentioned by 7 informants	2

Mentioned by 6 informants	0

Mentioned by 5 informants	1

Mentioned by 4 informants	8

Mentioned by 3 informants	9

Mentioned by 2 informants	23

Mentioned by 1 informant	54

**Table 7:** Ethnobotanical plants – Most mentioned species

**Species**	**Informants mentioning the species**
*Asparagus acutifolius *L.	12

*Beta vulgaris *L.	9

*Malva sylvestris *L.	7

*Opuntia ficus-indica *(L.) Miller	7

*Olea europaea *L. var. *europaea*	5

*Arbutus unedo *L.	4

*Cichorium intybus *L.	4

*Ferula communis *L.	4

*Foeniculum vulgare *Miller subsp. *piperitum *(Ucria) Coutinho	4

*Laurus nobilis *L.	4

*Mentha suaveolens *Ehrh. subsp. *insularis *(Req.) Greuter	4

*Parietaria diffusa *M. et K.	4

*Rubus ulmifolius *Schott	4

**Table 8:** Ethnobotanical plants – More versatile species

**species**	**number of secondary categories**	**number of categories**
*Asparagus acutifolius *L.	6	5

*Arbutus unedo *L.	5	4

*Malva sylvestris *L.	9	3

*Opuntia ficus-indica *(L.) Miller	4	3

*Cydonia oblonga *Miller	3	3

*Olea europaea *L. var. *sylvestris *Brot.	3	3

*Rubus ulmifolius *Schott	6	2

*Cichorium intybus *L.	5	2

*Crataegus monogyna *Jacq.	4	2

*Taraxacum officinale *Weber (s. l.)	4	2

*Lavatera cretica *L.	6	1

*Matricaria chamomilla *L.	5	1

**Table 9:** Ethnobotanical plants – Local frequency of species

	**Number of species**	**%**
Very common	53	*53,5*

Moderately common	36	*36,4*

Rare	9	*9,1*

Not present in the area	1	*1,0*

**Table 10:** Ethnobotanical plants – Habitats

	**Number of species**	**%**
Wastelands, marginal areas, courtyards, roadsides	58	58.6

Garrigues and shrubs	17	17.2

Wet and shady sites, near streams	14	14.1

Field edges, fences and hedges, dry walls	8	8.1

Cultivated landas, olive groves	8	8.1

Woods and maquis	6	6.1

Nearly everywhere	5	5.1

Mountain meadows	3	3

Rocks and rocky soils	3	3

North-facing mountainside	2	2

South-facing mountainside	1	1

Not present in the area	1	1

A total of 72 different plants were cited by the informants as being traditionally used in the area. These corresponded to 72 *ethnospecies *as defined in [18]. With approximately the same meaning, Berlin [22] suggests the use of the term *folk generic*; other authors, however, use different terms (see, for instance, Atran's *generic species *[23]). Based on a botanical identification of the specimens, these 72 ethnospecies correspond to 99 taxa (species or, in a few cases, subspecies) belonging to 83 genera and 34 families. Sixty-nine ethnospecies were identified by the informants with specific local names; three (corresponding to the botanical species *Plantago coronopus*, *Plantago lanceolata *and *Sanguisorba minor*) were recognized by the informants as a single ethnospecies, but no folk names were recorded. The rate of correspondence between folk names and scientific names was 81%. Nineteen percent of the folk names showed *under differentiation *(see [22]): i.e. a single ethnospecies name was used to indicate more than one botanical species (or subspecies) that sometimes even belonged to different botanical genera and families.

The families most mentioned by the informants (see Table 4) were Compositae (22 species, 57 citations), Liliaceae (7, 36), Rosaceae (7, 24, and Umbelliferae (6, 19). This is probably because of the abundance of these families in the Mediterranean flora, but it is also worth noting that they include many plants commonly used in Italy as food and natural medicine. Eighty-nine out of the 99 species grow wild, six are cultivated, three can be found either as wild or cultivated, and one (*Chamaerops humilis*) does not grow in the area under investigation and was usually collected in the surrounding areas (Table 5).

**Figure 1:**
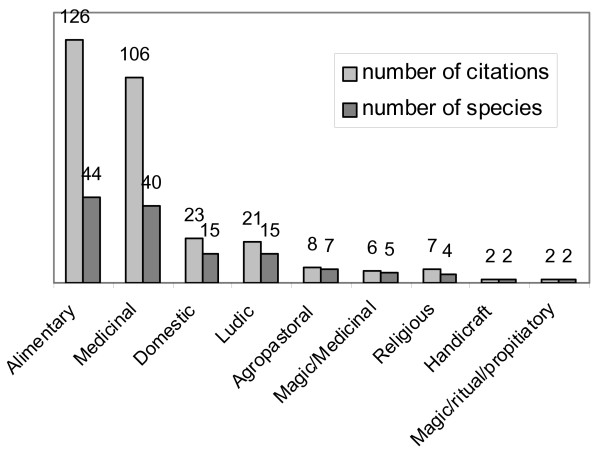
**Plants of ethnobotanical interest**. Categories of use.

A total of 301 citations was recorded, for 191 different uses belonging to 50 different secondary categories of use. The most relevant categories of use were Alimentary and Medicinal (Fig. 1)

Knowledge of the traditional uses of plants was heterogeneous and unevenly distributed among the informants (Table 6). Average values were 11.70 species and 18.59 uses known to each informant, but 70 species and 109 uses were reported by a single key-informant. Twenty-two species were mentioned by 3 or more informants. Out of these, only one was cited by more than 10 informants. This result probably reflects the disappearance of particular knowledge, rather than a low ethnobotanical value of the species cited by only one or two informants.

The species most mentioned (Table 7) were *Asparagus acutiflolius *(12 informants, 27 citations), *Beta vulgaris *(9, 16), *Malva sylvestris *(7, 19), *Opuntia ficus-indica *(7, 11), and *Olea europaea *var. *europaea *(5, 6). For most species (79%), only 1 to 2 different uses were reported. Species showing the highest versatility (Table 8), understood as being the highest number of different categories and secondary categories of use, were *Asparagus acutifolius *(5 different categories and 6 different secondary categories), *Arbutus unedo *(4 categories, 5 secondary categories), *Malva sylvestris *(3, 9), and *Opuntia ficus-indica *(3, 4). However, when only secondary categories of use were considered, *Rubus ulmifolius *(6 secondary categories, 2 categories), *Lavatera cretica *(6, 1), *Cichorium intybus *(5, 2), and *Matricaria chamomilla *(5, 1) were also found to be highly versatile.

As for the local frequency of species (Table 9), 53 species out of 99 (53.5%) were defined by the informants as 'very common', 36 (36.4%) as 'moderately common' and only 9 (9.1%) as 'rare' (one species was not present in the area).

**Figure 2:**
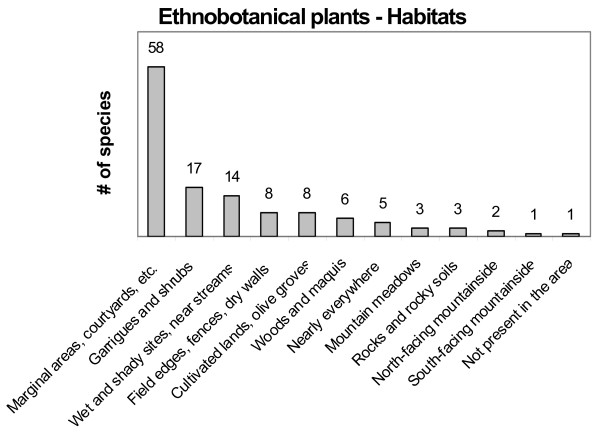
**Plants of ethnobotanical interest**. Gathering environments.

Plants of ethnobotanical interest were collected in 13 different environments (Table 10, Fig. 2). Ruderal species from wastelands, marginal areas, courtyards, and roadsides formed the largest portion (58.6%), followed by species growing in garrigues and shrubs (17.2%) and in wet and shady sites (14.1%). The predominance of the collection and consumption of species from environments marked and disturbed by human activity, rather than from less disturbed or pristine environments, is a common result in ethnobotanical studies, although few studies mention or discuss this point (e. g. [18,24,25]). Many hypotheses can be suggested as being possible explanations for this feature. First of all, people started recognizing and using wild plants that were easier to gather, such as those growing close to houses, roads, cultivated or fallow fields, and field boundaries. Many of the plants gathered in disturbed environments may also behave like weeds or opportunistic commensals in different agricultural practices, and have somehow co-evolved with the presence of man. It is also possible that some species of ethnobotanical interest were cultivated in the past and became wild afterwards.

It should also be noted that only 38 (41%) out of the 92 species growing wild in the area (including 3 species that are both wild and cultivated) were mentioned in 1957 in Rovinetti's phytogeographical contribution [26]. This can be explained in different ways. It is possible that, although he had been investigating in the area of Monte Ortobene for five years, Rovinetti failed to consider certain habitats, such as ruderal and marginal ones and ecotones, which are rich in plants of ethnobotanical interest. The possibility of floristic changes having taken place in past decades appears to be rather unlikely, since all the plants were cited as being traditionally used by old people.

Table 11 reports the results of quantitative analysis for the 20 most relevant and useful species in the area of Monte Ortobene.

**Table 11:** Results of quantitative analysis for the 20 most relevant species

	Basic values			Indices				Ranking		
**Species**	**FC**	**NC**	**UR**	**RFC**	**CI**	**H'**	**J**	**RFC**	**CI**	**H'**

*Asparagus acutifolius *L.	12	6	27	0.71	1.59	0.60	0.78	1	1	6

*Beta vulgaris *L.	9	3	16	0.53	0.94	0.38	0.79	2	3	13

*Malva sylvestris *L.	7	9	19	0.41	1.12	0.81	0.85	3	2	1

*Opuntia ficus-indica *(L.) Miller	7	4	9	0.41	0.53	0.57	0.95	4	6	7

*Olea europaea *L. var.*europaea*	5	3	6	0.29	0.35	0.44	0.92	5	9	11

*Rubus ulmifolius *Schott	4	6	10	0.24	0.59	0.76	0.98	6	5	2

*Arbutus unedo *L.	4	5	7	0.24	0.41	0.64	0.92	7	7	4

*Cichorium intybus *L.	4	5	11	0.24	0.65	0.64	0.91	8	4	5

*Laurus nobilis *L.	4	3	5	0.24	0.29	0.41	0.86	9	11	12

*Parietaria diffusa *Mert. et Koch	4	2	5	0.24	0.29	0.22	0.72	10	12	19

*Ferula communis *L.	4	2	5	0.24	0.29	0.22	0.72	11	13	18

*Foeniculum vulgare *Mill. subsp.*piperitum *(Ucria) Coutinho	4	2	7	0.24	0.41	0.30	0.99	12	8	14

*Mentha suaveolens *Ehrh. subsp.*insularis *(Req.) Greuter	4	2	4	0.24	0.24	0.24	0.81	13	14	17

*Matricaria chamomilla *L.	3	5	6	0.18	0.35	0.68	0.97	14	10	3

*Hedera helix *L.	3	3	4	0.18	0.24	0.45	0.95	15	15	10

*Daucus carota *L. subsp.*carota*	3	3	4	0.18	0.24	0.45	0.95	16	16	9

*Cydonia oblonga *Miller	3	3	4	0.18	0.24	0.45	0.95	17	17	8

*Rumex thyrsoides *Desf.	3	2	3	0.18	0.18	0.28	0.92	18	18	16

*Prunus spinosa *L.	3	2	3	0.18	0.18	0.28	0.92	19	19	15

*Allium triquetrum *L.	3	1	3	0.18	0.18	0.00	0.00	20	20	20

Basic values: FC = Number of informants mentioning the species; NC = Number of different uses ('secondary categories of use'); UR = Number of reports ('citations').

Indexes: RFC = Relative Frequency of Citation; CI = Cultural Importance Index; H'= Shannon-Wiener Index; J = Evenness index. (See 'Material and methods' for further explanations).

*Asparagus acutifolius *was the most used species (RFC = 0.71) and the most culturally significant (CI = 1.59). *Beta vulgaris *and *Malva sylvestris *were the second most important species, but the order varied, depending on the chosen index. The CI placed *Malva sylvestris *in second place, because it assigned a greater importance to the higher versatility of this species. On the other hand, the RFC took into consideration only the knowledge of useful plants, i.e. the number of informants who mentioned them as being useful ([19]), and *Beta vulgaris *was cited by more informants than *Malva sylvestris*. Table 11 also shows that *Asparagus acutifolius *and *Beta vulgaris *reached the 6^th ^(H' = 0.60) and 13^th ^(H' = 0.38) places, respectively, when the diversity of knowledge and its pattern of distribution within each species were considered. Moreover, *Rubus ulmifolius *was in 6^th ^place when only the number of informants was taken into account, (RFC = 0.24), but rose to 5^th ^place when the diversity of uses was considered (CI = 0.59) and to 2nd place on the basis of the Shannon-Wiener Index (H' = 0.76). The distribution of information was highly homogeneous for this species (Evenness = 0.98), and this can explain the high ranking based on the Shannon-Wiener Index. Due to the small number of informants, the results of the quantitative analysis were not as reliable as they could have been from a statistical point of view. However, these data were both encouraging and consistent with previous studies reporting on the use of synthetic indexes [19]. In our opinion, they could be valuable in a general discussion on the use of quantitative analysis in ethnobotanical research, and can be regarded as a pilot methodology for providing possible indications for further studies involving a larger number of informants (see, in particular, the use of the Shannon-Wiener Index to assess the diversity of uses within and between species).

In the following sections, results for each category of use are discussed. The categories are organized in order of abundance.

### Alimentary plants

A complete and detailed list of alimentary plants and uses is reported in Additional file 2.

For this category of use, 126 citations were recorded for 44 species and 13 different uses (secondary categories).

As expected, the botanical family most represented was Compositae, with 33 citations (26.2% of citations for this category) and 14 species (see Table 12). In order of abundance, these are followed by Liliaceae, with 23 citations (8.3%) and 5 species, Chenopodiaceae, with 15 citations but only one species, Umbelliferae (13 citations, 4 species), and Rosaceae (10 citations, 5 species). Families such as Cruciferae or Labiatae, which are usually very well represented in surveys on alimentary wild plants, had fewer citations.

**Table 12:** Alimentary plants – Botanical families

**Family**	**Citations**	***%***	**Species**
Compositae	33	*26.2*	14

Liliaceae	23	*18.3*	5

Chenopodiaceae	15	*11.9*	1

Umbelliferae	13	*10.3*	4

Rosaceae	10	*7.9*	5

Cruciferae	5	*4.0*	3

Boraginaceae	4	*3.2*	2

Ericaceae	4	*3.2*	1

Others (< 4 citations, 1 species)	19	*15.1*	9

When considering the number of species (21), the alimentary use most cited was, surprisingly, the one that we ourselves defined as a 'rural snack'. This means eating plants as soon as they are gathered, directly in the fields, outside of regular organized meals. This secondary category included plants eaten as fresh fruits or as raw vegetables (tender leaves or stems, the latter sometimes peeled: see picture in Additional file 3), and also flowers sucked by children in order to savour their sweet sugary nectar. This peculiar way of consuming wild plants has already been pointed out for other Mediterranean countries ([27]).

**Figure 3:**
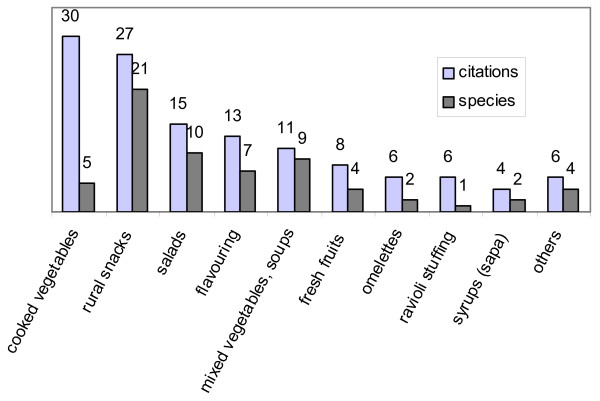
**Alimentary plants**. Secondary categories of use.

Plants eaten as cooked vegetables were found to be the most important with regard to the number of citations (30) (Fig. 3).

Six species received five or more citations, and most of these were wild relatives of cultivated plants. The species most mentioned was wild asparagus (*Asparagus acutifolius*) with 17 citations and 2 different uses (cooked vegetables, omelettes), followed by wild chard (*Beta vulgaris*) with 15 citations and 2 uses (cooked vegetables, ravioli stuffing), chicory (*Cichorium intybus*) with 8 citations and 3 uses (salads, cooked vegetables, a substitute for coffee), wild fennel (*Foeniculum vulgare *subsp. *piperitum*) with 7 citations and 2 uses (flavouring for canned olives, cooked vegetables), blackberry (*Rubus ulmifolius*) with 5 citations and 3 uses (rural snack, fresh fruits, jam), and *Sonchus tenerrimus *with 5 citations and 3 uses (raw salad, mixed vegetables, rural snack). Most of these plants are commonly used as wild food plants also in many other parts of Italy. Elsewhere, *Sonchus tenerrimus *is more frequently replaced by the more common, close species *Sonchus oleraceus*.

No species was mentioned by all informants. *Asparagus acutifolius *was cited by 12 informants (70.6% of the informants), *Beta vulgaris *was cited by 9 informants (52.9%), and all the remaining species were cited by less than 6 informants.

**Figure 4:**
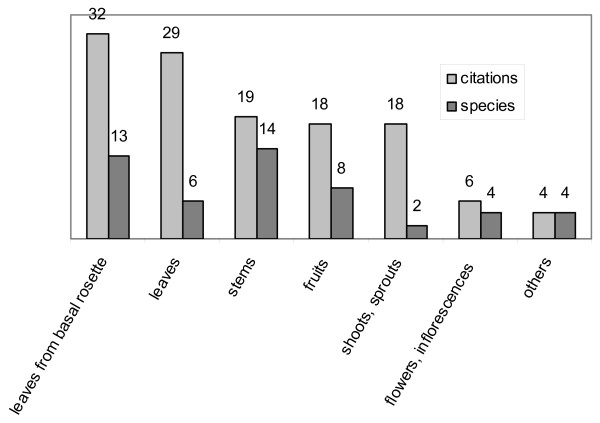
**Alimentary plants**. Parts used.

The most frequently used plant parts were leaves from basal rosettes (13 species, 32 citations) and other leaves (6 species, 29 citations), which are eaten mainly as mixed vegetables or raw salads. They were followed by stems (14 species, 19 citations), used as flavouring (*Foeniculum*, *Allium *species), eaten as raw salad, mixed vegetables or rural snack, and fruits (8 species, 18 citations), eaten fresh or dried or are used to prepare jams or a typical syrup (sapa) (Fig. 4).

All the species were easy to find and gather, and mostly grew very close to houses and cultivated fields. Twenty-seven alimentary species (61.4%) were defined by the informants as 'very common'; 13 species (29.5%) as 'moderately common', and only 4 (9.1%) as 'rare'. Most species (32) were found in ruderal anthropic habitats, such as wastelands, marginal areas, courtyards, roadsides. These were followed at a great distance by plants growing in garrigues and shrubs (6 species), wet and shady places (5) and other gathering environments.

According to many of the informants, most alimentary plants are still being gathered by people at the present time.

### Medicinal plants

For a detailed list of medicinal plants and uses, see Additional file 2.

In this category, 106 citations were recorded for 40 species and 12 different uses, namely as different general therapeutic indications.

Medicinal plants were found to belong to 23 botanical families (see Table 13). More than one quarter of all citations (24, i.e. 22.6% of all citations) involved Malvaceae, even if only 3 species were mentioned for this family. The next family was Compositae, with 18 citations (17%) and the highest number of species (6, i.e. 15% of medicinal species).

**Table 13:** Medicinal plants – Botanical families

**Family**	**Number of citations**	***% citations***	**Number of species**	***% species***
Malvaceae	24	*22,6*	3	*7,5*

Compositae	18	*17,0*	6	*15,0*

Rosaceae	11	*10,4*	5	*12,5*

Liliaceae	8	*7,5*	2	*5,0*

Urticaceae	6	*5,7*	3	*7,5*

Lauraceae	5	*4,7*	1	*2,5*

Oleaceae	5	*4,7*	1	*2,5*

Cactaceae	4	*3,8*	1	*2,5*

others (< 4 citations, 1–2 species)	25	*23,6*	18	*45,0*

**Figure 5:**
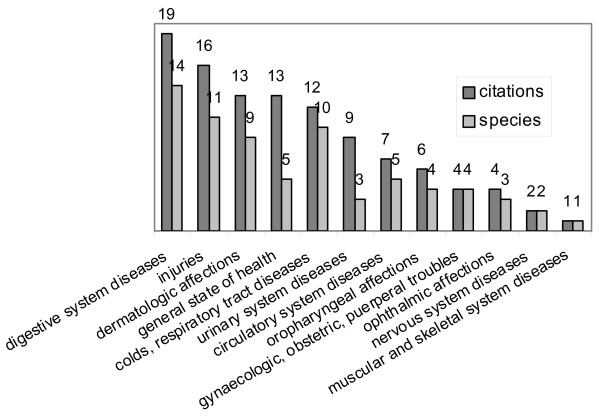
**Medicinal plants**. Secondary categories of use.

The most cited medicinal uses concerned diseases of the digestive system (21 species, 56 citations), followed by injuries (wounds, burns, stings, animals bites, etc.), with 11 species and 16 citations, dermatological affections (9 species, 13 citations), and general state of health (5 species, 13 citations) (Fig. 5). This last secondary category included plants regarded as effective, not against a specific affection, but in curing and strengthening the body as a whole. Some of these plants are defined as 'cleansing', 'depurant' or 'detoxicant', meaning that they can help in recovering from different diseases by purifying the blood and removing toxins and scum, and possibly by stimulating diuresis. Others are called 'refreshers' or 'anti-inflammables', meaning that they can be effective in relieving pain in the different apparatuses, thanks to their general soothing action and perhaps also by acting as mild laxatives. Such generic and somehow indefinite therapeutic indications are common in ethnobotany, and can be regarded as signs of genuine information (see also [[18,28] pp. 300, 301]).

*Malva sylvestris *(17 citations, 7 informants) and *Asparagus acutifolius *(7 citations, 7 informants) were found to be the best known and most used medicinal species. Mallow was also found to be the most versatile species (7 different therapeutic indications), while wild asparagus was mentioned only for one use (as a diuretic). *Lavatera cretica *received 6 citations for 6 different uses, but all of them were recorded by only one informant. Indeed, most of the species (27) were mentioned by only one informant, thus revealing that knowledge is unevenly distributed within the community.

**Figure 6:**
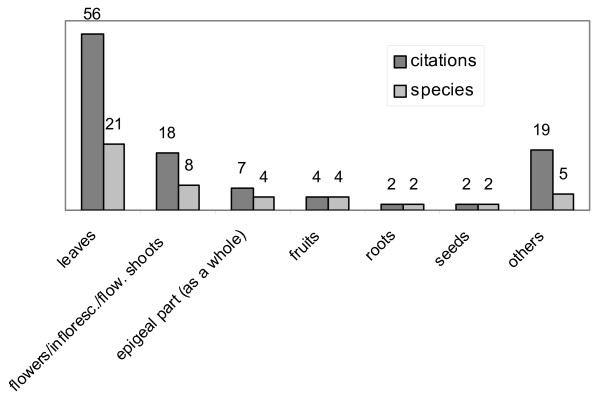
**Medicinal plants**. Parts used.

The most used plant parts are leaves (56 citations, 21 species), followed by flowers and inflorescences (18 citations, 8 species), whole epigeal parts (7, 4), and fruits (4, 4) (Fig. 6). Note that the prevailing role of leaves is a habitual result in ethnobotanical investigations on medicinal plants carried out in Italy and Europe (see, for instance, [8,13,29,30]), while in other parts of the world – for example, in many developing countries – this is not necessarily the case.

**Figure 7:**
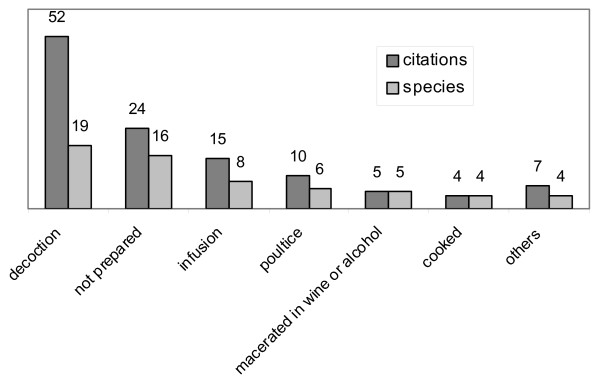
**Medicinal plants**. Ways of preparation.

**Figure 8:**
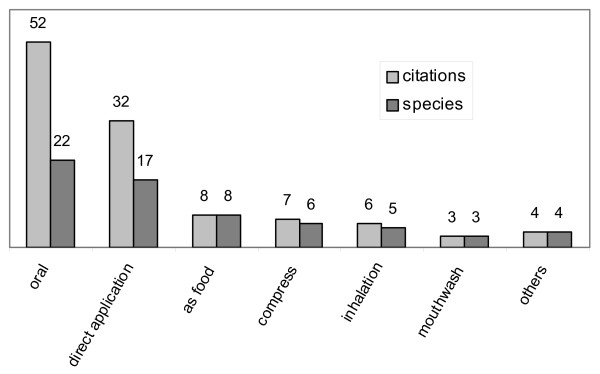
**Medicinal plants**. Ways of administration.

Remedies are mostly prepared as decoctions (52 citations, 19 species), or used without being prepared (24, 16), prepared as infusions (15, 8) or as poultices (10, 6) (Fig. 7). Oral dosing, direct application, and giving as food are the most frequently recorded ways of administration (Fig. 8). Simplicity in preparing and administering remedies is regarded as a sign of genuine information (see, among others, [21,31,32]).

Most of the species were reported by the informants as being 'very common' on Monte Ortobene (24 species, i.e. 60%); 11 species (27.5%) were considered 'moderately common', and only 5 species (12.5%) were termed 'rare'. Almost all the medicinal species (34, i.e. 85%) grow wild in the area; 4 (10%) are cultivated, and 2 (5%) can be found both as wild and as cultivated plants.

Similarly to the alimentary plants reported on, medicinal species are mostly found in ruderal or disturbed habitats (22 species, 55%).

Most medicinal plants were reported as having been used in the past, but as not being used any more by the community.

### Other categories

Not many plants and uses were recorded for the remaining categories of use. A short discussion follows, but see Additional file 2 for details.

#### Domestic

Fifteen species were recorded as being traditionally used in this category, with 23 citations for eight different secondary categories of use.

Plants were mainly used for body care, more precisely for rinsing and curing hair. Other uses involved fireplaces and chimneys (twigs used to light a fire or to clean chimneys), scenting rooms or linens, plants used for dyeing clothes or brightening up their colours, repellents for moths or mice, and to make brooms. One informant reported the peculiar use of young shoots of wild olive as a whip to punish children. The most recorded species was *Lavandula stoechas *(4 citations), used both to scent linens and as a repellent against moths.

#### Ludic

In this category, plants are used – mostly by children – to play games, jokes and as pastimes, or to make toys, dolls and ornaments or fancy-dress costumes. Fifteen species were recorded, with 21 citations for five different secondary categories of use. See Additional file 2 for more information and Additional file 3 for pictures.

#### Agropastoral

Very few plants and uses were recorded for this category (7 species, 8 citations, 5 uses). *Opuntia *branches are planted to serve as fences; cut-off branches of other thorny plants are placed above typical dry-stone walls (*sa cresura*) to keep people and/or animals out. Plants are also used in sheep farming, pork butchering, wine producing, and to make certain agricultural tools.

#### Magical/Medicinal

For this category, six citations were recorded, for five species and two different uses. While the magical use of plants to heal warts is frequent in many parts of Italy, the rite for treating jaundice reported by one informant is very peculiar. Jaundice is known locally as *su male 'e s'istria *(which means 'barn owl's disease'), because the coming of a barn owl is believed to portend this illness. Four different plants (*Artemisia arborescens*, *Helychrysum italicum *and blessed olive and palm tree leaves), together with salt and holy water, are involved in this complicated rite, which must be performed by a healer and repeated for three days (see Additional file 2 for details).

Indeed, it should also be noted that the use of many medicinal plants also includes some magical aspects in collecting the plants and/or in preparing and administering the remedies.

#### Religious

Seven citations were recorded for this category. Four plants were reported as being used to decorate altars on various feast days (Christmas, Easter, Corpus Christi). See Additional file 3 for images.

#### Handicrafts

Only two citations for two ethnobotanical uses (stools and baskets) were mentioned by the informants for this category. See Additional file 3 for images.

#### Magical/ritual/propitiatory

Two traditional magical uses of plants were recorded: a propitiatory agricultural practice to keep birds away from sown fields, and predictions regarding future husbands for young women.

### Further information

During the investigation, further information was also collected. It is reported here in a separate section, either because the data are incomplete, or because they are not of strictly ethnobotanical interest (e. g. traditional cookies prepared with almonds, which come from cultivated trees and are normally used in this way).

#### Ornamental plants

A few informants reported the usage of picking up wild flowers to decorate houses. The following species were mentioned:

*Cyclamen repandum *S. et S.

*Dactylorhiza insularis *(Sommier) Landwehr

*Lupinus angustifolius *L.

*Ophrys incubacea *Bianca

*Orchis longicornu *Poiret

*Orchis papilionacea *L.

*Petrorhagia velutina *(Guss) P.W. Ball & Heywood

*Ranunculus *cfr. *monspeliacus *L.

In fact, it is very likely that many other plants are commonly collected for this purpose – perhaps all those with colored or attractive flowers – and possibly even the same informants would have mentioned different species if they had been interviewed in a different season.

#### Toxic plants

The following plants were pointed out as being poisonous or potentially toxic for humans or livestock:

*Arbutus unedo *L. (eating too many fruits may cause nausea and vomiting)

*Asparagus acutifolius *L. (people suffering from diabetes, hypertension or renal troubles are advised not to eat too many sprouts by way of vegetables)

*Ferula communis *L. (this plant is poisonous for sheep)

*Papaver rhoeas *L. (the excessive use of seeds as a soporific for babies may cause convulsions)

#### Traditional biscuits

Some biscuits traditionally prepared with almonds for special festivities were reported by a few informants, together with recipes and related customs. One of these customs (see: *papassinos*) is particularly interesting, as it is very similar to the traditional American celebration of Halloween.

##### Guerfos

Prepared with sweet and bitter almonds (finely chopped), sugar, honey, lemon peel and some brandy. All the ingredients are combined together and cooked until the mixture is thick enough to make small, compact spheres that will be sprinkled with icing sugar. The same mixture is used to stuff a candied citrus fruit called *pompia. Guerfos*, which are typically made for baptisms and weddings, are nowadays prepared and consumed for no special occasion, but throughout the year. For traditional weddings, the bridegroom used to bring to the bride's house 12 hearts made of pastry stuffed with the *guerfos *mixture.

##### Zarminu

Similar to meringues, these cookies are prepared with egg whites beaten with sugar. The batter is shaped into small rounds with toasted and sliced almonds at the centre of each round, then put in the oven to bake. Before serving, the *zarminu *are trimmed with small silver-colored ornaments called *trazzea*. They are typically made for parties and receptions.

##### Papassinos

Prepared with water, wheat flour, lard, almonds, walnuts and raisins, mixed together. The pastry is rolled and cut into small rhombs that are baked in the oven, then iced with a mixture of egg whites and sugar, and lastly trimmed with colored *trazzea*.

These are made typically for All Souls' Day (November 2^nd^). The evening before All Souls, children visit the neighbouring houses knocking on doors and ask, '*Su mortu, mortu*?' (i.e. 'Is the dead dead?'). In the past, housewives replied to this request by giving almonds, walnuts, dried figs, pomegranates, quinces or *papassinos *to the children; nowadays, the fruits and sweets have been replaced by coins.

##### Sa arantzada

Prepared with orange peel, boiled, cut into small strips and mixed with heated honey and almonds. This mixture is also used to stuff the candied citrus called *pompia*. Typically prepared for baptism parties.

##### Amarettos

Sweet almonds are chopped, and are then mixed with egg whites, flour, sugar and a few bitter almonds. The pastry is shaped into round biscuits, which are then baked in the oven. Prepared for weddings, baptisms and important festivities.

#### Sayings

*Allium triquetrum *(in local language: apara).

There is a traditional joking metaphor: 'S'este falau chie s'apara' (i. e.: 'It wilted like wild garlic'), referring to 'something' that, after 'blooming', looses all vigour and appeal. In fact, the inflorescence of this plant quickly wilts in the presence of heat or when picked.

*Reichardia picroides *(in local language: mammalucca).

The words *mammalucca *(for women) and *mammaluccu *(for men) are also used to designate stupid people.

## Conclusion

The information collected at Monte Ortobene and presented here shows that a certain richness and diversity of knowledge regarding traditional uses for plants still survives as a part of the cultural heritage of the community. However, this knowledge is ageing, and is likely to vanish fairly soon. As reported by most of the informants, many practices are no longer in use and survive only as memories from the past. Even on the basis of a small number of informants, this pattern is particularly clear in medicinal uses (see, for instance, the large number of plants cited by fewer than 3 informants), and is confirmed by the results of the quantitative analyses (Table 11). It is clear that these traditional phytotherapeutic remedies were more popular and widespread in the past, when medicines were not easily available. At present it is not surprising that, for the sake of their own health, people have a greater faith in 'official' medicines prescribed by doctors than they do in folk remedies. On the contrary, several edible wild plants are still gathered and processed by members of the community, and many of these plants are popular both as delicacies and as the ingredients of local specialties.

In general, in the Monte Ortobene area as in other Mediterranean zones, the younger generations have lost much of the traditional knowledge necessary for identifying, gathering and treating wild plant species. It is thus becoming crucial that this biocultural diversity be recorded and preserved by means of proper documentation and an identification of the relative species before it vanishes definitively. It is also of great importance to consider this cultural heritage within the framework of a sustainable management approach, with the aim of preserving all the components of its environmental diversity.

## Competing interests

The authors declare that they have no competing interests.

## Authors' contributions

MAS and MP conceived the research. MP carried out the interviews, collected all the data in the field, and arranged them in the first version of the analytical table. MAS and PB processed the data and drafted the manuscript together. PB performed the quantitative analysis. All authors read and approved the final manuscript.

## Supplementary Material

Additional file 1 **List of species**. List of plants of ethnobotanical interest, supplemented with some additional information. Click here for file

Additional file 2 **Categories of use: lists of plants and uses**. The sheet contains information about plants and uses for each category of use. Within each category, the plants are grouped together according to the secondary category of use, and short descriptions are given on how each plant is used. Click here for file

Additional file 3 **Additional figures**. Images showing some traditional uses of plants in Monte Ortobene. Click here for file
