# Supplementary material for: Plants and traditional knowledge: An ethnobotanical investigation on Monte Ortobene (Nuoro, Sardinia)
Source: J Ethnobiol Ethnomed. 2009 Feb 10;5:6. doi: 10.1186/1746-4269-5-6 (PMC2661884; doi:10.1186/1746-4269-5-6)
Supplement: Additional file 1 — List of species. List of plants of ethnobotanical interest, supplemented with some additional information. [file 1746-4269-5-6-S1.pdf]

Additional file 1. Ethnobotanical investigation on Monte Ortobene (Sardinia, Italy). List of species of ethnobotanical interest

Species	Botanical family	Folk names	FC	Used parts	Categories of use	Number of categories of use	Number of different uses (secondary categories)	Number of citations	Wild/ cultivated w: wild c: cultivated w/c: both	Local frequency r: rare mc: moderately common vc: very common	RFC	CI	H'	J
Achillea ligustica All.	Compositae	Arculentu	2	Inflorescences	Medicinal	1	2	3	w	vc	0,12	0,18	0,28	0,92
Allium ampeloprasum L.	Liliaceae	Azu	1	Bulbs	Alimentary	1	1	1	w	r	0,06	0,06	0,00	0,00
Allium subhirsutum L.	Liliaceae	Azu	1	Herbaceous stems	Alimentary	1	1	1	w	mc	0,06	0,06	0,00	0,00
Allium triquetrum L.	Liliaceae	Apara	3	Herbaceous stems	Alimentary	1	1	3	w	mc	0,18	0,18	0,00	0,00
Arbutus unedo L.	Ericaceae	Lidone	4	Fruits, wood	Alimentary, Domestic, Ludic, Medicinal	4	5	7	w	r	0,24	0,41	0,64	0,92
Artemisia arborescens L.	Compositae	Assentu	2	Flowers, epigeal part	Magic/ medicinal, Medicinal	2	2	2	w	vc	0,12	0,12	0,30	1,00
Arundo donax L.	Graminaceae	Canna	1	Stems	Agropastoral, Ludic	2	2	2	w	vc	0,06	0,12	0,30	1,00
Asparagus acutifolius L.	Liliaceae	Isparagu, ispina sorichina	12	Shoots or sprouts, epigeal part	Agropastoral, Alimentary, Domestic, Medicinal, Religious	5	6	27	w	mc	0,71	1,59	0,60	0,78
Asparagus albus L.	Liliaceae	Isparagu	1	Shoots or sprouts	Alimentary	1	1	1	w	r	0,06	0,06	0,00	0,00
Asphodelus microcarpus Salzm. et Viv.	Liliaceae	Irbuttu	2	Leaves, roots	Ludic, Medicinal	2	2	2	w	vc	0,12	0,12	0,30	1,00
Ballota nigra L. subsp. *uncinata* (Fiori et Bèg.) Patzak	Labiatae	Marrubiu nigheddu	1	Leaves	Medicinal	1	1	1	w	mc	0,06	0,06	0,00	0,00

Species	Botanical family	Folk names	FC	Used parts	Categories of use	Number of categories of use	Number of different uses (secondary categories)	Number of citations	Wild/ cultivated w: wild c: cultivated w/c: both	Local frequency r: rare mc: moderately common vc: very common	RFC	CI	H'	J
Bellis perennis L.	Compositae	Margherited da	1	Inflorescences	Ludic	1	1	1	w	vc	0,06	0,06	0,00	0,00
Bellis sylvestris Cyr.	Compositae	Margherited da	1	Inflorescences	Ludic	1	1	1	w	vc	0,06	0,06	0,00	0,00
Beta vulgaris L.	Chenopodiacea e	Beda	9	Leaves	Alimentary, Medicinal	2	3	16	w	vc	0,53	0,94	0,38	0,79
Borago officinalis L.	Boraginaceae	Suzzamele	1	Leaves from basal rosette, flowers	Alimentary	1	2	2	w	vc	0,06	0,12	0,30	1,00
Briza maxima L.	Graminaceae	Sonaiolos	1	Inflorescences	Ludic	1	1	1	w	vc	0,06	0,06	0,00	0,00
Calendula arvensis L. subsp. *arvensis*	Compositae	Frore de cada mese	1	Epigeal part (as a whole), inflorescences	Medicinal	1	2	2	w	vc	0,06	0,12	0,30	1,00
Calicotome villosa (Poiret) Link	Leguminosae	Tiria	1	Branches/ twigs	Agropastoral	1	1	1	w	mc	0,06	0,06	0,00	0,00
Capsella bursa-pastoris (L.) Medicus	Cruciferae	Isperra carzones	1	Epigeal part (as a whole)	Medicinal	1	1	1	w	vc	0,06	0,06	0,00	0,00
Carduus cephalanthus Viv.	Compositae	Gardu	1	Stems	Alimentary	1	1	1	w	vc	0,06	0,06	0,00	0,00
Carduus pycnocephalus L.	Compositae	Gardu	1	Stems	Alimentary	1	1	1	w	vc	0,06	0,06	0,00	0,00
Carthamus lanatus L.	Compositae	Gardu	1	Stems	Alimentary	1	1	1	w	vc	0,06	0,06	0,00	0,00
Centaurea calcitrapa L.	Compositae	Gardu	1	Inflorescences (bracts)	Agropastoral	1	1	1	w	mc	0,06	0,06	0,00	0,00
Chamaerops humilis L.	Palmae	palma	1	Leaves	Magic / medicinal	1	1	1	-		0,06	0,06	0,00	0,00
Chondrilla juncea L.	Compositae	Su porru	1	Herbaceous stems	Alimentary	1	3	3	w	vc	0,06	0,18	0,48	1,00

Species	Botanical family	Folk names	FC	Used parts	Categories of use	Number of categories of use	Number of different uses (secondary categories)	Number of citations	Wild/ cultivated w: wild c: cultivated w/c: both	Local frequency r: rare mc: moderately common vc: very common	RFC	CI	H'	J
Cichorium intybus L.	Compositae	Zicoria	4	Leaves from basal rosette, roots	Alimentary, Medicinal	2	5	11	w	vc	0,24	0,65	0,64	0,91
Cistus albidus L.	Cistaceae	Mudrecu	1	Branches/twigs	Domestic	1	1	1	w	mc	0,06	0,06	0,00	0,00
Cistus salvifolius L.	Cistaceae	Mudrecu	1	Branches/twigs	Domestic	1	1	1	w	mc	0,06	0,06	0,00	0,00
Crataegus monogyna Jacq.	Rosaceae	Calabriche	2	Flowers, fruits	Alimentary, Medicinal	2	4	4	w	mc	0,12	0,24	0,60	1,00
Crepis vesicaria L.	Compositae	Zicoria burda	2	Leaves from basal rosette, flowers	Alimentary	1	2	4	w	vc	0,12	0,24	0,30	1,00
Cydonia oblonga Miller	Rosaceae	Mela chidonza	3	Fruits	Alimentary, Domestic, Medicinal	3	3	4	c	mc	0,18	0,24	0,45	0,95
Daucus carota L. subsp. *carota*	Umbelliferae	Frustinaca agreste	3	Leaves from basal rosette, roots	Alimentary	1	3	4	w	vc	0,18	0,24	0,45	0,95
Daucus carota L. subsp. *maximum* (Desf.) Ball.	Umbelliferae	Frustinaca agreste	1	Leaves from basal rosette	Alimentary	1	1	1	w	vc	0,06	0,06	0,00	0,00
Echium plantagineum L.	Boraginaceae	Suzzamele	2	Flowers	Alimentary	1	1	2	w	vc	0,12	0,12	0,00	0,00
Eucalyptus globulus Labill.	Myrtaceae	Eucaliptu	2	Leaves	Medicinal	1	1	2	c	r	0,12	0,12	0,00	0,00
Euphorbia helioscopia L.	Euphorbiaceae	Lattoricu	1	Latex	Medicinal	1	1	1	w	vc	0,06	0,06	0,00	0,00
Ferula communis L.	Umbelliferae	Ferula	4	Stems	Handicraft, Ludic	2	2	5	w	mc	0,24	0,29	0,22	0,72

Species	Botanical family	Folk names	FC	Used parts	Categories of use	Number of categories of use	Number of different uses (secondary categories)	Number of citations	Wild/ cultivated w: wild c: cultivated w/c: both	Local frequency r: rare mc: moderately common vc: very common	RFC	CI	H'	J
Foeniculum vulgare Miller subsp. *piperitum* (Ucria) Coutinho	Umbelliferae	Frinucu	4	Leaves, stems	Alimentary	1	2	7	w	vc	0,24	0,41	0,30	0,99
Galactites tomentosa Moench	Compositae	Gardu mele	2	Stems	Alimentary	1	1	2	w	vc	0,12	0,12	0,00	0,00
Halimium halimifolium (L.) Willk	Cistaceae	Mudrecu	1	Branches/twig s	Domestic	1	1	1	w	mc	0,06	0,06	0,00	0,00
Hedera helix L.	Araliaceae	Edera	3	Leaves	Domestic, Medicinal	2	3	4	w	vc	0,18	0,24	0,45	0,95
Helichrysum italicum (Roth) Don	Compositae	Uscradinu, frore de Santu Jubanne	2	Epigeal part (as a whole)	Agropastoral, Magic/ medicinal	2	2	2	w	vc	0,12	0,12	0,30	1,00
Hyoseris radiata L.	Compositae	Paparanzolu	1	Herbaceous stems	Alimentary	1	1	1	w	r	0,06	0,06	0,00	0,00
Hypericum perforatum L.	Guttiferae	Pericu	2	Flowering shoots	Medicinal	1	2	3	w	mc	0,12	0,18	0,28	0,92
Hypochoeris radicata L.	Compositae	Zicoria burda	2	Herbaceous stems, leaves from basal rosette	Alimentary	1	2	2	w	mc	0,12	0,12	0,30	1,00
Juncus inflexus L.	Juncaceae	Zuncu	2	Leaves	Magic/ medicinal	1	1	2	w	r	0,12	0,12	0,30	0,00
Lagurus ovatus L.	Graminaceae	Mussitos	1	Epigeal part (as a whole)	Ludic	1	1	1	w	vc	0,06	0,06	0,00	0,00
Laurus nobilis L.	Lauraceae	Labru	4	Leaves	Medicinal	1	3	5	c	mc	0,24	0,29	0,41	0,86
Lavandula stoechas L.	Labiatae	Archimissa	2	Inflorescences	Domestic	1	2	4	w	mc	0,12	0,24	0,30	1,00
Lavatera cretica L.	Malvaceae	Marmaruzza	1	Leaves	Medicinal	1	6	6	w	vc	0,06	0,35	0,78	1,00
Lavatera olbia L.	Malvaceae	Marmara	1	Flowers	Medicinal	1	1	1	w	vc	0,06	0,06	0,00	0,00

Species	Botanical family	Folk names	FC	Used parts	Categories of use	Number of categories of use	Number of different uses (secondary categories)	Number of citations	Wild/ cultivated w: wild c: cultivated w/c: both	Local frequency r: rare mc: moderately common vc: very common	RFC	CI	H'	J
Malva parviflora L.	Malvaceae	Su panicheddu	1	Fruits	Alimentary	1	1	1	w	vc	0,06	0,06	0,00	0,00
Malva sylvestris L.	Malvaceae	Marmaruzza	7	Leaves, roots	Domestic, Ludic, Medicinal	3	9	19	w	vc	0,41	1,12	0,81	0,85
Marrubium vulgare L.	Labiatae	Marrubiu	1	Leaves	Medicinal	1	1	1	w	mc	0,06	0,06	0,00	0,00
Matricaria chamomilla L.	Compositae	Camomilla	3	Inflorescences	Medicinal	1	5	6	w	r	0,18	0,35	0,68	0,97
Medicago arabica (L.) Hudson	Leguminosae	Trivozu	1	Leaves	Medicinal	1	2	2	w	vc	0,06	0,12	0,30	1,00
Mentha suaveolens Ehrh. subsp. *insularis* (Req.) Greuter	Labiatae	Mentastru	4	Leaves, live plants	Alimentary, Domestic	2	2	4	w	mc	0,24	0,24	0,24	0,81
Myrtus communis L.	Myrtaceae	Murta	2	Leaves, fruits	Alimentary	1	2	3	w/c	mc	0,12	0,18	0,28	0,92
Nerium oleander L.	Apocynaceae	Neulache	1	Leaves	Medicinal	1	1	1	w/c	mc	0,06	0,06	0,00	0,00
Olea europea L. var. *sylvestris* Brot.	Oleaceae	Ozzastru	1	Wood, young twigs	Handicraft, Domestic, Ludic	3	3	3	w	mc	0,06	0,18	0,48	1,00
Olea europea L. var. *europaea*	Oleaceae	Oliba	5	Leaves, oil, seeds	Magic/ medicinal, Medicinal	2	3	6	c	mc	0,29	0,35	0,44	0,92
Opuntia ficus-indica (L.) Miller	Cactaceae	Ficu murisca	7	Fruits, lymph, live plants	Agropastoral, Alimentary, Medicinal	3	4	9	w	vc	0,41	0,53	0,57	0,95
Papaver rhoeas L.	Papaveraceae	Attanda	2	Flowers, seeds	Ludic, Medicinal	2	2	2	w	vc	0,12	0,12	0,30	1,00
Parietaria diffusa M. et K.	Urticaceae	Erba e bentu	4	Epigeal part (as a whole)	Alimentary, Medicinal	2	2	5	w	vc	0,24	0,29	0,22	0,72
Pinus pinaster Aiton	Pinaceae	Pinu	1	Leaves, wood	Ludic	1	2	2	w	mc	0,06	0,12	0,30	1,00

Species	Botanical family	Folk names	FC	Used parts	Categories of use	Number of categories of use	Number of different uses (secondary categories)	Number of citations	Wild/ cultivated w: wild c: cultivated w/c: both	Local frequency r: rare mc: moderately common vc: very common	RFC	CI	H'	J
Pistacia lentiscus L.	Anacardiaceae	Chessa	3	Bark	Medicinal	1	1	3	w	vc	0,18	0,18	0,00	0,00
Plantago coronopus L.	Plantaginaceae	_	1	Leaves from basal rosette, stems	Alimentary	1	3	3	w	vc	0,06	0,18	0,48	1,00
Plantago lanceolata L.	Plantaginaceae	_	1	Leaves from basal rosette	Medicinal	1	1	1	w	vc	0,06	0,06	0,00	0,00
Prunus dulcis (Miller) D.A. Webb	Rosaceae	Mendula	1	Fruits (endocarps)	Medicinal	1	1	1	w/c	mc	0,06	0,06	0,00	0,00
Prunus spinosa L.	Rosaceae	Prunischedda , Isprunazza	3	Fruits, branches	Agropastoral, Alimentary	2	2	3	w	mc	0,18	0,18	0,28	0,92
Pyrus amygdaliformis Vill.	Rosaceae	Pirastru	1	Fruits	Alimentary	1	1	1	w	mc	0,06	0,06	0,00	0,00
Quercus pubescens Willd.	Fagaceae	Chercu	2	Galls	Ludic	1	2	3	w	mc	0,12	0,18	0,28	0,92
Ranunculus bulbosus L.	Ranunculaceae	Ranunculu	1	Leaves	Medicinal	1	1	1	w	r	0,06	0,06	0,00	0,00
Raphanus raphanistrum L.	Cruciferae	Ermulatta	3	Leaves from basal rosette	Alimentary	1	1	3	w	vc	0,18	0,18	0,00	0,00
Reichardia picroides (L.) Roth	Compositae	Mammalucca	1	Leaves from basal rosette	Alimentary	1	2	2	w	mc	0,06	0,12	0,30	1,00
Rubus ulmifolius Schott	Rosaceae	Rubu	4	Leaves, fruits	Alimentary, Medicinal	2	6	10	w	vc	0,24	0,59	0,76	0,98
Rumex thyrsoides Desf.	Polygonaceae	Miliacra	3	Leaves	Alimentary	1	2	3	w	vc	0,18	0,18	0,28	0,92
Sambucus nigra L.	Caprifoliaceae	Sabucu	1	Flowers	Medicinal	1	1	1	w	mc	0,06	0,06	0,00	0,00
Sanguisorba minor Scop.	Rosaceae	_	1	Leaves	Medicinal	1	1	1	w	r	0,06	0,06	0,00	0,00
Scandix pecten-veneris L.	Umbelliferae	Orolozeddos	1	Fruits	Ludic	1	1	1	w	vc	0,06	0,06	0,00	0,00
Scolymus hispanicus L.	Compositae	Gardu	1	Stems	Alimentary	1	1	1	w	vc	0,06	0,06	0,00	0,00

Species	Botanical family	Folk names	FC	Used parts	Categories of use	Number of categories of use	Number of different uses (secondary categories)	Number of citations	Wild/ cultivated w: wild c: cultivated w/c: both	Local frequency r: rare mc: moderately common vc: very common	RFC	CI	H'	J
Sedum caeruleum L.	Crassulaceae	Achinedda e monte	1	Leaves	Alimentary	1	1	1	w	vc	0,06	0,06	0,00	0,00
Silybum marianum (L.) Gaertner	Compositae	Gardu	1	Stems	Alimentary	1	1	1	w	vc	0,06	0,06	0,00	0,00
Sinapis arvensis L.	Cruciferae	Ermulata	1	Leaves from basal rosette	Alimentary	1	1	1	w	mc	0,06	0,06	0,00	0,00
Sisymbrium officinale (L.) Scop.	Cruciferae	Ermulata	1	Leaves from basal rosette	Alimentary	1	1	1	w	mc	0,06	0,06	0,00	0,00
Smilax aspera L.	Liliaceae	Teti	1	Fruits	Domestic	1	1	1	w	mc	0,06	0,06	0,00	0,00
Smyrnium rotundifolium Miller	Umbelliferae	Brentedda	1	Stems	Alimentary	1	1	1	w	vc	0,06	0,06	0,00	0,00
Sonchus tenerrimus L.	Compositae	Gardu minzone, zicoria burda	2	Leaves from basal rosette	Alimentary	1	3	5	w	vc	0,12	0,29	0,46	0,96
Taraxacum officinale Weber (s. l.)	Compositae	Zicoria burda	1	Leaves from basal rosette	Alimentary, Medicinal	2	4	4	w	vc	0,06	0,24	0,45	0,75
Teline monspessulana (L.) Koch	Leguminosae	Matricusa	2	Branches/ twigs	Domestic	1	2	3	w	mc	0,12	0,18	0,28	0,92
Triticum durum Desf.	Graminaceae	Tridicu	2	Live plants (etiolated)	Magic/ritual/ propitiatory, Religious	2	2	3	c	mc	0,12	0,18	0,28	0,92
Umbilicus horizontalis (Guss.) DC.	Crassulaceae	Caliche e muru	2	Leaves	Ludic, Medicinal	2	3	3	w	vc	0,12	0,18	0,48	1,00
Urtica dioica L.	Urticaceae	Pistiddori	1	Leaves	Medicinal	1	1	1	w	vc	0,06	0,06	0,00	0,00
Urtica membranacea L.	Urticaceae	Pistiddori	1	Epigeal part (as a whole)	Domestic, Medicinal	2	2	2	w	vc	0,06	0,12	0,00	0,00
Urtica urens L.	Urticaceae	Pistiddori	1	Epigeal part (as a whole)	Domestic	1	1	1	w	vc	0,06	0,06	0,00	0,00

Species	Botanical family	Folk names	FC	Used parts	Categories of use	Number of categories of use	Number of different uses (secondary categories)	Number of citations	Wild/ cultivated w: wild c: cultivated w/c: both	Local frequency r: rare mc: moderately common vc: very common	RFC	CI	H'	J
Verbascum pulverulentum Vill.	Scrophulariaceae	Trovodda	1	Live plants	Magic/ritual/ propitiatory	1	1	1	w	vc	0,06	0,06	0,00	0,00
Vicia villosa Roth	Leguminosae	Pane e casu	2	Flowers	Alimentary	1	1	2	w	vc	0,12	0,12	0,00	0,00
Vinca difformis Pourret	Apocynaceae	Pruinca	2	Epigeal part (as a whole)	Religious	1	1	2	w	mc	0,12	0,12	0,00	0,00
Vinca major L. var. *variegata* Pourret	Apocynaceae	Pruinca	2	Epigeal part (as a whole)	Religious	1	1	2	c	mc	0,12	0,12	0,00	0,00
