# Supplementary material for: Plants and traditional knowledge: An ethnobotanical investigation on Monte Ortobene (Nuoro, Sardinia)
Source: J Ethnobiol Ethnomed. 2009 Feb 10;5:6. doi: 10.1186/1746-4269-5-6 (PMC2661884; doi:10.1186/1746-4269-5-6)
Supplement: Additional file 2 — Categories of use: lists of plants and uses. The sheet contains information about plants and uses for each category of use. Within each category, the plants are grouped together according to the secondary category of use, and short descriptions are given on how each plant is used. [file 1746-4269-5-6-S2.pdf]

Additional file 2. List of plants of ethnobotanical interest, supplemented with some additional information

Species	*Use description*	*Informants mentioning this use*
**ALIMENTARY PLANTS**		
**Cooked vegetables**		
Asparagus acutifolius L.	Turions are boiled and dressed with olive oil and salt, or sauté with olive oil.	12
Beta vulgaris L.	Leaves are boiled and dressed with olive oil and salt.	9
Cichorium intybus L.	Leaves from the basal rosette are boiled and dressed with olive oil and salt, or sauté with bacon and lard.	3
Crepis vesicaria L.	Leaves are boiled and dressed with olive oil and salt.	2
Foeniculum vulgare Miller subsp. *piperitum* (Ucria) Coutinho	Leaves from young shoots are boiled and dressed with olive oil and salt, or sauté, or cooked with broad beans and pork rinds to prepare a dish called *fave e lardu.*	4
**Rural snacks**		
Borago officinalis L.	The sweet nectar from freshly collected flowers is sucked, especially by children.	1
Carduus cephalanthus Viv.	Tender stems are peeled and eaten as fresh vegetables in the fields.	1
Carduus pycnocephalus L.	Tender stems are peeled and eaten as fresh vegetables in the fields.	1
Carthamus lanatus L.	Tender stems are peeled and eaten as fresh vegetables in the fields.	1
Chondrilla juncea L.	Eaten in the fields as fresh vegetables.	1
Daucus carota L. subsp. *carota*	Tender leaves are eaten as fresh vegetables in the fields.	2
Daucus carota L. subsp. *maximum* (Desf.) Ball.	Tender leaves are eaten as fresh vegetables in the fields.	1
Echium plantagineum L.	The sweet nectar from freshly collected flowers is sucked, especially by children.	2
Galactites tomentosa Moench	Tender stems are peeled and eaten as fresh vegetables in the fields.	2

Hyoseris radiata L.	The inflorescence scape is eaten when still tender, in the fields. The presence of latex gives it a sweetish taste.	1
Malva parviflora L.	The sweet-tasting fruit, shaped as a small loaf, is eaten by children.	1
Plantago coronopus L.	Young shoots are eaten as fresh vegetables in the fields.	1
Reichardia picroides (L.) Roth	Leaves are eaten as fresh vegetables in the fields, especially by children.	1
Rubus ulmifolius Schott	Fruits are eaten fresh in the fields.	1
Rumex thyrsoides Desf.	Tender leaves are eaten as fresh vegetables in the fields, especially by children.	2
Scolymus hispanicus L.	Leaves, small and succulent, are eaten as fresh vegetables in the fields.	1
Sedum caeruleum L.	Leaves, small and succulent, are eaten as fresh vegetables in the fields.	1
Silybum marianum (L.) Gaertner	Tender stems are peeled and eaten as fresh vegetables in the fields.	1
Smyrnium rotundifolium Miller	Tender stems are peeled and eaten as fresh vegetables.	1
Sonchus tenerrimus L.	The young leaves are eaten both in salads and directly in the fields.	2
Vicia villosa Roth	The sweet nectar from freshly collected flowers is sucked, especially by children.	2
**Salads**		
Chondrilla juncea L.	Leaves are used in mixed salads, dressed with olive oil and salt.	1
Cichorium intybus L.	Leaves are used as salads, dressed with olive oil and salt.	4
Crepis vesicaria L.	Leaves are used in salads (often mixed with other vegetables, to mitigate their bitterish taste), dressed with olive oil and salt.	2
Daucus carota L. subsp. *carota*	Leaves are used as salads, dressed with olive oil and salt.	1
Hypochoeris radicata L.	Leaves and young, tender shoots are used in salads with other herbs.	1
Plantago coronopus L.	Used in salads with other herbs.	1
Reichardia picroides (L.) Roth	Tender leaves are used in mixed salads.	1

Rumex thyrsoides Desf.	Leaves are used in mixed salads.	1
Sonchus tenerrimus L.	Leaves are used as salads.	2
Taraxacum officinale Weber (s. l.)	Used in salads with other herbs.	1
**Flavouring**		
Allium ampeloprasum L.	Bulbs are used to flavour various dishes.	1
Allium subhirsutum L.	Minced stems are used to flavour salads.	1
Allium triquetrum L.	Minced stems are used in salads (to give them the typical wild onion flavour), or sauté in olive oil before frying eggs in it.	3
Cydonia oblonga Miller	Fresh fruits are used in the syrup *sapa de ficu murisca* (see below).	1
Daucus carota L. subsp. *carota*	Used to flavour game meat, with other herbs such as parsley and celery.	1
Foeniculum vulgare Miller subsp. *piperitum* (Ucria) Coutinho	Green stems (gathered in autumn) are used to flavour vegetable soups or the water in which olives are preserved.	3
Mentha suaveolens Ehrh. ssp. *insularis* (Req.) Greuter	Leaves are used to flavour a kind of black pudding called *su zurrette* or *su sambene*, prepared with sheep's blood, *carasau* bread soaked in milk, pecorino cheese, bacon or lard, basil and *Mentha suaveolens*, boiled in a sheep's stomach and served on *carasau* bread.	3
**Mixed vegetables and soups**		
Borago officinalis L.	Boiled with other herbs, as *Beta*.	1
Chondrilla juncea L.	Used to prepare vegetable soups.	1
*Hypochoeris radicat*a L.	Leaves from the basal rosette are cooked with other herbs.	1
Parietaria diffusa M. et K.	Mashed with potatoes and other vegetables.	1
Plantago coronopus L.	Boiled (sometimes with other herbs), dressed with olive oil and salt.	1
Raphanus raphanistrum L.	Basal leaves are boiled, then sauté with olive oil, lard or bacon. Eaten as a full meal.	3
Sinapis arvensis L.	Boiled and eaten as cooked vegetables or sauté with lard.	1
Sisymbrium officinale (L.) Scop.	Boiled and eaten as cooked vegetables or sauté with lard.	1
Sonchus tenerrimus L.	Boiled with *Beta* and other herbs, dressed with olive	1

	oil and salt.	
**Fresh fruits**		
Arbutus unedo L.	Berries are eaten as fresh fruits.	3
Prunus spinosa L.	Fruits are left to wither on the branches until autumn before eating them; in this way they become less sour and more palatable.	2
Pyrus amygdaliformis Vill.	Fruits are eaten when fully ripened and dark-coloured.	1
Rubus ulmifolius Schott	Fruits are eaten directly when ripe.	2
**Omelettes**		
Asparagus acutifolius L.	Turions are boiled and used in omelettes.	5
Asparagus albus L.	Tender turions, deprived of their thorns, are boiled and used in omelettes.	1
**Ravioli stuffing**		
Beta vulgaris L.	Leaves are boiled, minced and mixed with ricotta cheese to fill ravioli called *culurjones.*	6
**Syrups (*sapa*)**		
Arbutus unedo L.	Berries are boiled in water and pressed; the filtered liquid is boiled until it thickens. This syrup, called *sapa de mela 'e lidone,* is used in traditional confectionery. *Sapa* is usually made with grape-must, but also other fruits can be used.	1
Opuntia ficus-indica (L.) Miller	Prickly pears, washed (to remove thorns) but not peeled, are boiled with quinces and orange peels; the filtered liquid, with the addition of sugar, is boiled again until it thickens. This syrup, called *sapa de ficu murisca*, can be thickened with semolina to obtain a kind of jam, eaten with bread or used to fill traditional cakes; it can be preserved for a long time (even for 10 years).	3
**Liqueurs**		
Myrtus communis L.	Leaves or berries are soaked in alcohol; the filtered liquid, with the addition of water and sugar, is used to prepare a liqueur.	2
**Jams**		
Rubus ulmifolius Schott	Fruits are used to prepare jam.	2
**Drinks**		
Cichorium intybus L.	Roots, dried and ground, are used as a substitute for	1

	coffee.	
**Pickles**		
Crataegus monogyna Jacq.	Flower buds are cooked, pickled and eaten as capers.	1
**MEDICINAL PLANTS**		
**Oropharyngeal affections**		
Lavatera cretica L.	Bleeding gums: gargles with a decoction of leaves.	1
Malva sylvestris L.	Swollen gums: a poultice of fresh leaves or gargles with a decoction of leaves.	3
Matricaria chamomilla L.	Sore throat: an infusion of inflorescences, filtered and sweetened with honey, taken orally.	1
Rubus ulmifolius Schott	Bleeding gums: gargles with a decoction of apical leaves from shoots.	1
**Dermatologic affections**		
Calendula arvensis L. subsp. *arvensis*	Warts: the whole plant is crushed and applied locally.	1
Euphorbia helioscopia L.	Warts: the latex is applied locally.	1
Hypericum perforatum L.	Skin rashes: the flowering shoots are macerated in olive oil, in sunlight, for 40 days; the liquid is filtered and applied locally.	1
Lavatera cretica L.	Furuncles: the leaves are boiled in water and squeezed or used fresh, oiled with lard, and applied locally as a poultice until maturation.	1
Malva sylvestris L.	Furuncles: the leaves are boiled in water and squeezed or used fresh, oiled with lard, and applied locally as a poultice until maturation. Sometimes the skin is treated with olive oil before applying the poultice. Dandruff, corns: a poultice prepared as above is applied locally.	3
Medicago arabica (L.) Hudson	Furuncles: fresh leaves are crushed and applied locally.	1
Nerium oleander L.	Purulent skin eruptions: a decoction of leaves is applied as a compress. *Herpes zoster* eruptions ('Fuoco di sant'Antonio'): as above, or immersion of the body part (if a limb) in a decoction of leaves.	1

Opuntia ficus-indica (L.) Miller	Sores, rhagas, skin mycoses ('athlete's foot'): the lymph from stems is applied locally; the stems are peeled or split in two and heated in the oven. Sometimes the split, heated stem is applied directly.	3
Umbilicus horizontalis (Guss.) DC.	Sores: fresh leaves are split in two to let the gelatinous lymph out and applied locally.	1
**Ophthalmic affections**		
Lavatera cretica L.	Conjunctivitis: a decoction of leaves is applied as a compress.	1
Malva sylvestris L.	Conjunctivitis: a decoction of leaves is applied as a compress.	1
Matricaria chamomilla L.	Conjunctivitis: a decoction of inflorescences is applied as a compress.	2
**Circulatory system diseases**		
Asphodelus microcarpus Salzm. et Viv.	Hemorrhoids: the fresh root is split in two and applied locally.	1
Crataegus monogyna Jacq.	An infusion of dry flowers is drunk as a cardiotonic.	1
Olea europaea L. var. *europaea*	High blood pressure: a decoction (100 leaves boiled for 30 minutes and filtered, or 20 leaves boiled for 15 minutes) is taken orally (a small glass a day for 7/10 days). Hemorrhoids: the seeds are pounded, mixed with olive oil, wrapped in a gauze and applied locally as a poultice.	2
Rubus ulmifolius Schott	Hemorrhoids: the shoots are boiled, wrapped in a cloth and applied as a poultice; a decoction of leaves can be used in the same way.	2
Taraxacum officinale Weber (s. l.)	High levels of cholesterol: a decoction of leaves from the basal rosette is taken orally.	1
**Digestive system diseases**		
Achillea ligustica All.	Poor digestion: drink a small glass of inflorescences soaked in wine for 30 days. Gastric ulcer pain: a teaspoon of dried inflorescences a day, taken orally.	2
Artemisia arborescens L.	Poor digestion: a pinch of dried flower heads or an infusion (for 10 minutes) of flower heads is taken orally.	1
Ballota nigra L. subsp. *uncinata* (Fiori et Bèg) Patzak	Poor digestion: soaked in wine for 20-40 days, then taken orally.	1

Beta vulgaris L.	Constipation: the leaves, boiled with Malva sylvestris leaves and dressed with olive oil, vinegar and salt, are taken as food.	1
Cichorium intybus L.	Liver trouble: an infusion of leaves is taken orally. Fresh leaves can also be eaten as a salad.	1
Crataegus monogyna Jacq.	Diarrhoea: the fresh fruit is eaten as food.	1
Laurus nobilis L.	Poor digestion, stomach-ache: a small glass of wine in which leaves have been soaked for a week, or a decoction in water and sugar of 12-15 leaves, or an infusion of leaves are taken orally.	3
Malva sylvestris L.	Stomach-ache: a decoction of roots (boiled in water until it becomes of a yellowish colour, then filtered) is taken orally. Constipation: the leaves, boiled with Beta vulgaris leaves and dressed with olive oil, vinegar and salt, are taken as food.	2
Marrubium vulgare L.	Poor digestion: the leaves are macerated in wine for 20-40 days, then taken orally. An infusion of leaves, sweetened with sugar of honey, is used in the same way.	1
Matricaria chamomilla L.	Stomach-ache: an infusion of inflorescences is taken orally.	1
Myrtus communis L.	Poor digestion: a decoction of leaves sweetened with sugar or honey, or a liqueur prepared with leaves, are taken orally.	1
Rubus ulmifolius Schott	Ulcer: a filtered decoction of young shoots (1 kg for 1 l of water, boil until the water is reduced to half the volume, can be kept in the fridge for 10 days) is taken orally (a small glass a day for 15/30 days, half an hour before breakfast). Stomach-ache: the decoction is taken when necessary.	2
Taraxacum officinale Weber (s. l.)	Poor digestion: boiled leaves are eaten as food. Liver trouble: the decoction has a choleretic action (stimulates bile production from the gall bladder) and is taken orally.	1
Urtica dioica L.	Ulcer: a decoction of leaves (10 minutes, then filter) is taken orally.	1
**Muscular and skeletal system diseases**		

Ranunculus bulbosus L.	Sciatica: the leaves are lightly crushed and applied directly on the heel (in absence of abrasions) for a night, using plastic film as a cover; the day after the resulting blister is perforated, cutting the skin all around to remove both the serum and the pain.	1
**Urinary system diseases**		
Arbutus unedo L.	As a diuretic: fresh fruits are eaten as food.	1
Asparagus acutifolius L.	As a diuretic: a decoction of turions or the water used for cooking them.	7
Hedera helix L.	Cellulitis: a decoction of leaves is directly applied as a compress.	1
**Gynaecologic diseases, obstetric and puerperal troubles**		
Calendula arvensis L. subsp. *arvensis*	Menstrual pain: a decoction of inflorescences (a handful in a cup of water) is taken orally during the 10 days preceding the period.	1
Capsella bursa-pastoris (L.) Medicus	Irregular menstrual cycles: an infusion of the epigean parts is taken orally during the 10 days preceding the period.	1
Laurus nobilis L.	Menstrual pain: an infusion of few leaves with Matricaria chamomilla inflorescences is taken orally.	1
Matricaria chamomilla L.	Menstrual pain: an infusion of inflorescences with few *Laurus nobilis* leaves is taken orally.	1
**Nervous system diseases**		
Crataegus monogyna Jacq.	Anxiety disorders: an infusion of dried flowers is taken orally.	1
Papaver rhoeas L.	Sleep difficulties in babies: a few seeds are wrapped in a handkerchief and used as a dummy.	1
**Colds, respiratory tract diseases**		
Cydonia oblonga Miller	Colds, catarrhs: the decoction (one fruit boiled in water for 15 minutes with five dried figs and a *Laurus nobilis* leaf) can be used for inhalations or eaten as food (fruits and all), sweetened with two tablespoons of honey.	1
Eucalyptus globulus Labill.	Colds, catarrhs, coughs: an infusion of leaves is used for inhalations.	2
Laurus nobilis L.	Colds, catarrhs: the decoction (one leaf boiled in water for 15 minutes with five dried figs and a fruit of *Cydonia oblonga*) can be used for inhalations or eaten as food (fruits and all), sweetened with two tablespoons of honey.	1
Lavatera cretica L.	Colds, asthma: a decoction of leaves (boiled with *Sambucus nigra* leaves) is used for inhalations.	1

Lavatera olbia L.	Colds, coughs: a decoction of flowers (boil for 10 minutes, filter), sweetened with honey, is taken orally.	1
Malva sylvestris L.	Colds, asthma: a decoction of leaves, boiled with *Sambucus nigra* leaves, is used for inhalations. Colds, coughs: a decoction of leaves sweetened with honey is taken orally.	2
Matricaria chamomilla L.	Catarrhs: an infusion di inflorescences sweetened with honey is taken orally.	1
Prunus dulcis (Miller) D.A. Webb	Colds: a decoction di endocarps (fruits' shells) is taken orally. A handful of shells is boiled for 20 minutes, then filtered, sweetened with honey and drunk slowly.	1
Sambucus nigra L.	Colds: a filtered decoction of flowers is taken orally.	1
Urtica membranacea L.	Bronchitis: the leaves are boiled for a few minutes, then applied on the chest as a poultice under a woollen sweater.	1
**General state of health**		
Cichorium intybus L.	As a depurant-detoxicant: a decoction of leaves (boiled for 10 minutes) or the water used for cooking leaves are taken orally, preferably in the morning.	2
Lavatera cretica L.	As a 'refresher' (anti-inflammatory): a decoction of leaves, sweetened with sugar or honey, is taken orally.	1
Malva sylvestris L.	As a 'refresher' (anti-inflammatory): a filtered decoction of leaves, sometimes sweetened with sugar or honey, is taken orally.	5
Parietaria diffusa M. et K.	As a 'refresher' (anti-inflammatory): a filtered decoction of leaves (boiled for a few minutes), sometimes sweetened with sugar or honey, is taken orally.	4
Taraxacum officinale Weber (s. l.)	As a 'depurant': a decoction of leaves is taken orally.	1
**Injuries**		
Achillea ligustica All.	Small wounds: fresh inflorescences are applied locally as a cicatrizant.	1
Hypericum perforatum L.	Scalds, burns, solar erythema, wounds: the 'oleolito' (flowering shoots soaked in olive oil in sunlight for 20-40 days, then filtered) is applied locally as a lenitive and cicatrizant.	2
Lavatera cretica L.	Sores: a decoction of leaves is used for cleaning.	1
Malva sylvestris L.	Sores: a decoction of leaves is used for cleaning.	1
Medicago arabica (L.) Hudson	Small wounds: fresh, crushed leaves are applied locally as an haemostatic	1

Olea europaea L. var. *europaea*	Sunburns: an emulsion of olive oil and water is appled locally as a lenitive. Sprains and dislocations: the aching part is massaged with warm oil. Venomous stings and bites: the oil in which a live gecko ('Tattaruledda') was soaked is applied locally.	3
Opuntia ficus-indica (L.) Miller	Burns, abrasions: the lymph taken from stems is applied locally as a lenitive and cicatrizant. The stems, split in two and oiled, are heated in the oven or in the foreplace.	1
Pistacia lentiscus L.	Wounds: fresh bark is scraped off from a branch, pulverized and applied locally as a cicatrizant.	3
Plantago lanceolata L.	Wounds: fresh, crushed leaves are applied locally as an haemostatic and cicatrizant.	1
Sanguisorba minor Scop.	Wounds: fresh, minced leaves are applied locally as an haemostatic	1
Umbilicus horizontalis (Guss.) DC.	Wounds: the gelatinous lymph extracted from leaves is applied locally as a cicatrizant.	1
**PLANTS FOR DOMESTIC USE**		
**Body care products and cosmetics**		
Hedera helix L.	A decoction of leaves is used to rinse dark hair, adding colour and strength	2
Malva sylvestris L.	A decoction of leaves is used to rinse hair, making them soft.	1
Urtica membranacea L.	A decoction of leaves is used to rinse hair, making them soft and bright and preventing dandruff and loss of hair.	1
Urtica urens L.	A decoction of leaves is used to rinse hair, making them soft and bright and preventing dandruff and loss of hair.	1
**For fireplaces and chimneys**		
Asparagus acutifolius L.	Branches are rolled up in a ball used to clean chimneys.	1
Cistus albidus L.	Branches are used as tinder.	1
Cistus salvifolius L.	Branches are used as tinder.	1
Halimium halimifolium (L.) Willk	Branches are used as tinder.	1
Teline monspessulana (L.) Koch	Branches are used as tinder.	1
**To scent rooms or linen**		
Cydonia oblonga Miller	Fruits are put upon furniture to scent rooms, or between linen items to perfume them.	2
Lavandula stoechas L.	Flowers are put inside wardrobes, often in small cloth bags, as a perfume.	2
**Repellents**		

Lavandula stoechas L.	Flowers are put inside wardrobes, often in small cloth bags, both as a perfume and against moths.	2
Mentha suaveolens Ehrh. ssp. *insularis* (Req.) Greuter	Planted around houses to keep mice away.	1
**Dyes**		
Arbutus unedo L.	Colour: orange. Berries are boiled in water, then left to soak until the colour oozes out; clothes are boiled in the filtered liquid for 20 minutes.	1
Smilax aspera L.	Colour: red. Berries are boiled in water, then squeezed; clothes are boiled in the filtered liquid for 20 minutes. The intensity of colour may vary depending on how many berries are used.	1
**Detergents/dyes**		
Hedera helix L.	Fresh leaves are boiled for a long time; the dark-coloured water so obtained is used (with a soft brush) to clean dark clothes.	1
**Brooms**		
Teline monspessulana (L.) Koch	Small brooms are made from dried branches and used to clean ovens.	2
**Other uses**		
Olea europaea L. var. *sylvestris* Brot.	Young shoots are used to make whips called *frunzitta*, to punish vivacious children.	1
**PLANTS FOR LUDIC USE**		
**Dolls**		
Asphodelus microcarpus Salzm. et Viv.	A leaf is used for the doll's bust	1
Bellis perennis L.	The 'flowers' (inflorescences) are used for the doll's face	1
Bellis sylvestris Cyr	The 'flowers' (inflorescences) are used for the doll's face	1
Malva sylvestris L.	A leaf is used for the doll's skirt	1
**Toys**		
Arbutus unedo L.	The wood is used to make the *bardoffula*, a spinning top with a nail for a point. The toy is wound up in string that will be used to make it spin when thrown on the ground. A circle is drawn on the ground and each player will try to break the others' tops or to throw them outside the circle with his one, or to make his top spin for the longest time.	1
Arundo donax L.	Used to make horse-shaped toys.	1
Ferula communis L.	Little boys use this plant to make carts.	4

Olea europaea L. var. *sylvestris* Brot.	The wood is used to make the *bardoffula* (see *Arbutus)*	1
*Pinus pinaste*r Aiton	The wood is used to make the *bardoffula* (see *Arbutus)*	1
Quercus pubescens Willd.	Galls, pierced with small sticks, are used as spinning tops	1
**Group games and jokes**		
Papaver rhoeas L.	The game consists of guessing the petals' colour before opening the flower bud	1
Quercus pubescens Willd.	Galls are used as marbles and pushed with the thumb. To win, a player must be the first to get the marbles at the end or a fixed track (without leaving it) or be able to throw them into a hole.	2
**Ornaments and fancy dresses for children**		
Lagurus ovatus L.	Two plants, twisted together, are used as moustaches and tied behind the ears.	1
Pinus pinaster Aiton	Leaves are tied in chains to make necklaces.	1
**Pastimes**		
Briza maxima L.	Inflorescences are shaken close to the ear to hear the sound of the spikelets.	1
Scandix pecten-veneris L.	The fruit is taken away and the carpophore is turned around in the light.	1
Umbilicus horizontalis (Guss.) DC.	The leaves are used as a drawing sheet, often with a *Prunus* thorn for pencil. The round, cup -shaped leaves so decorated are then sometimes offered to a relative or friend of the family to get sweets or dried fruits in exchange for them.	1
**PLANTS FOR AGROPASTORAL USE**		
**Fences, boundaries**		
Calicotome villosa (Poiret) Link	Thorny cut-off branches are placed above dry-stone walls (called *sa cresura*) to keep people or animals out.	1
Opuntia ficus-indica (L.) Miller	Planted to serve as fences, thanks to its thorny cladophylls.	2
Prunus spinosa L.	Thorny cut-off branches are placed above dry-stone walls (called *sa cresura*) to keep people or animals out.	1
**Others**		
Arundo donax L.	Used to make a tool for gathering prickly pears called *su boddidore*: the tip of a long reed is split into three-four parts, kept apart by inserting a stone and tied with rope. The fruit is caught in this fork and picked applying a slight torsion.	1

Centaurea calcitrapa L.	Thorny bracts are stuck into the flesh of slaughtered sheep to keep flies from laying eggs in it.	1
Helychrysum italicum (Roth) Don	A sheaf of dried plants is lit and used to burn pig's bristles, to flavour bacon, ham and meat.	1
Asparagus acutifolius L.	The plant, called *ispina sorichina*, is rolled up in a ball, then put against the hole in the tank where grapes are pressed, to act as a filter.	1
**PLANTS FOR MAGIC/MEDICINAL USE**		
**To treat *su male 'e s'istria* (the 'barn owl's disease')**, i.e. jaundice. It is believed that the coming of the barn owl portends this illness. To perform the magical rite the plants are put in an old terracotta tile with salt and holy water, then a flame is lighted inside the tile to diffuse smoke. The fingernails, the toenails and a lock of hair of the sick person are cut and put in the tile. Arm span and standing height of the sick person are measured. If the two measurements differ s'istria (the barn owl) has cast the spell; the smoking tile is placed near the sick one and the healer jumps over it making the sign of the cross for three times and saying a special prayer. Recovery will follow if the rite is performed for three days.		
Artemisia arborescens L.		1
Chamaerops humilis L.	The etiolated leaves are cut into ribbons and blessed in church on Palm Sunday, together with olive branches.	1
Helychrysum italicum (Roth) Don		1
Olea europaea L. var. *europaea*	Branches are blessed in church on Palm Sunday.	1
**To heal warts**		
Juncus inflexus L.	On a leaf are tied as many knots as there are warts; the leaf is then broken or thrown into a river (or stream) in a place where the sick person will never go. The warts will then disappear in a short time.	2
**PLANTS FOR RELIGIOUS USE**		
**Plants used to decorate altars on feasts**		
Asparagus acutifolius L.	Branches are gathered before the Christmas novena, decorated with small tufts of cotton wool and put upon the altar as an ormament.	1
Triticum durum Desf.	Plants are put to germinate on wet cotton wool upon a plate and kept in the dark for 40 days. The plates are then decorated with coloured paper and brought into church on the Thursday before Easter to decorate the Holy Sepulchre.	2
Vinca difformis Pourret	Branches with both leaves and flowers are used to decorate the arch above the Madonna statue on Corpus Christi Day.	2

Vinca major L. var. *variegata* Pourret	Branches with both leaves and flowers are used to decorate the arch above the Madonna statue on Corpus Christi Day.	2
**PLANTS FOR HANDICRAFT USE**		
Ferula communis L.	Cube-shaped stools are made with pieces of dried stems laid one on top of the other and tied with cords, or nailed.	1
Olea europaea L. var. *sylvestris* Brot.	The young, tender and flexible branches are used in winter to make small baskets called *pischeddu*.	1
**PLANTS FOR MAGIC/RITUAL/PROPITIATORY USE**		
Triticum durum Desf.	The plates of etiolated plants used to decorate the Holy Sepulchre on Thursday before Easter are placed on the corners of sown fields to keep birds away.	1
Verbascum pulverulentum Vill	On the evening of St. John's Day (June 24) each girl chooses a plant and marks it with a lace or string. The insects found upon the plant on the morning after symbolize the future husband's line of work: ant = farmer; beetle = merchant; winged ant = shepherd, and so on.	1
