# Supplementary material for: Plants and traditional knowledge: An ethnobotanical investigation on Monte Ortobene (Nuoro, Sardinia)
Source: J Ethnobiol Ethnomed. 2009 Feb 10;5:6. doi: 10.1186/1746-4269-5-6 (PMC2661884; doi:10.1186/1746-4269-5-6)
Supplement: Additional file 3 — Additional figures. Images showing some traditional uses of plants in Monte Ortobene. [file 1746-4269-5-6-S3.ppt]

## Slide 1
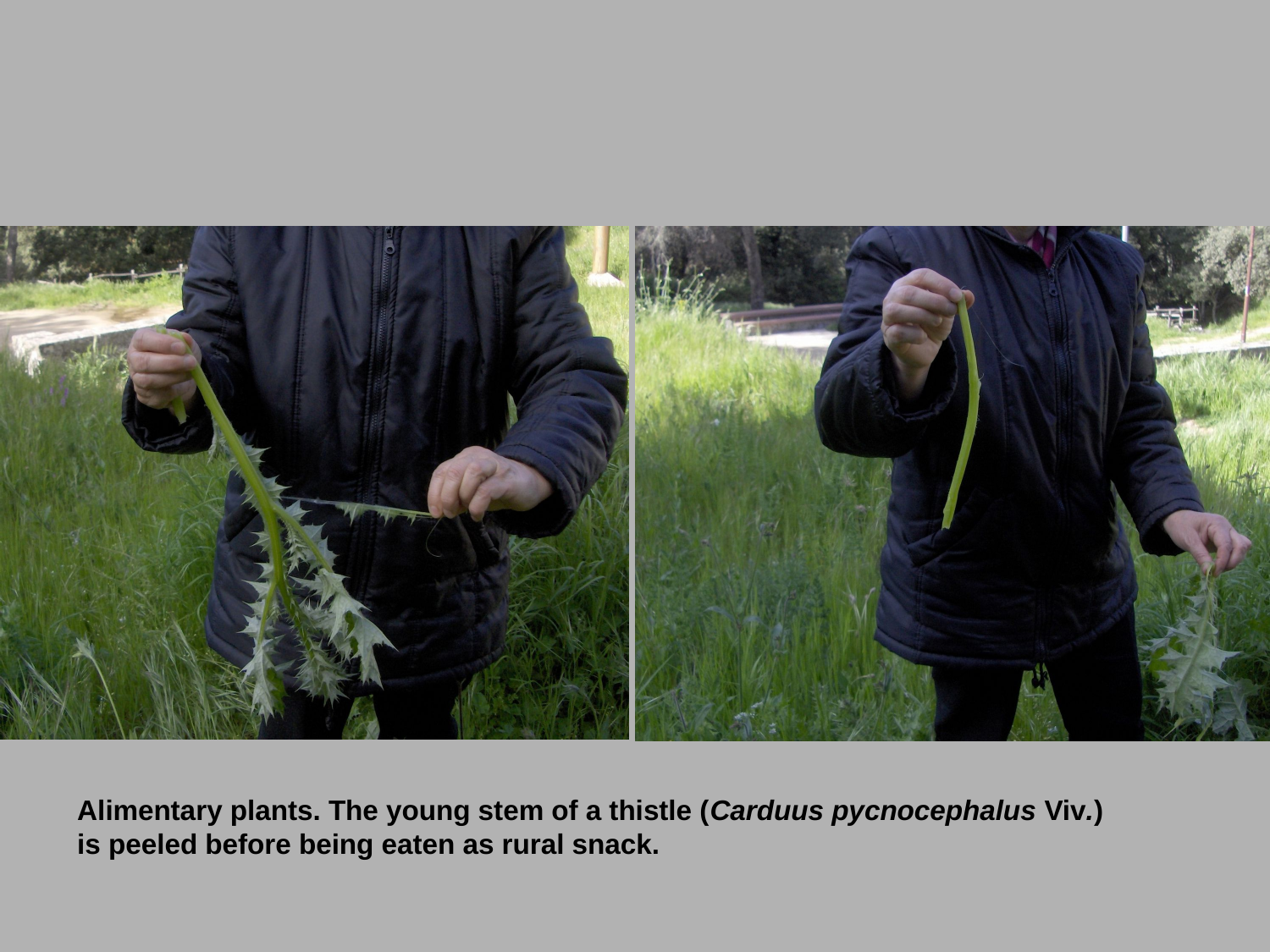

Alimentary plants. The young stem of a thistle (Carduus pycnocephalus Viv.) is peeled before being eaten as rural snack.

## Slide 2
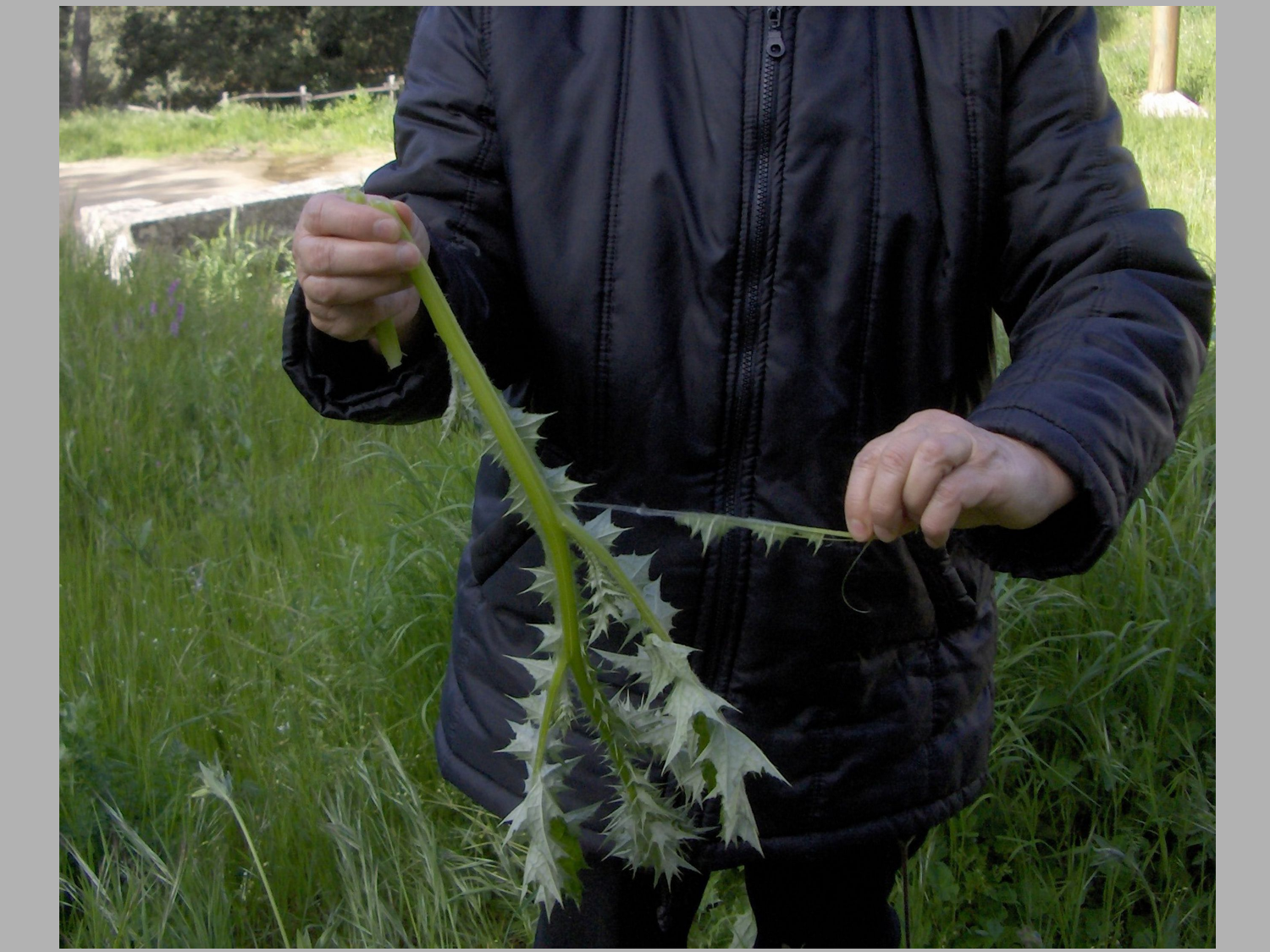

## Slide 3
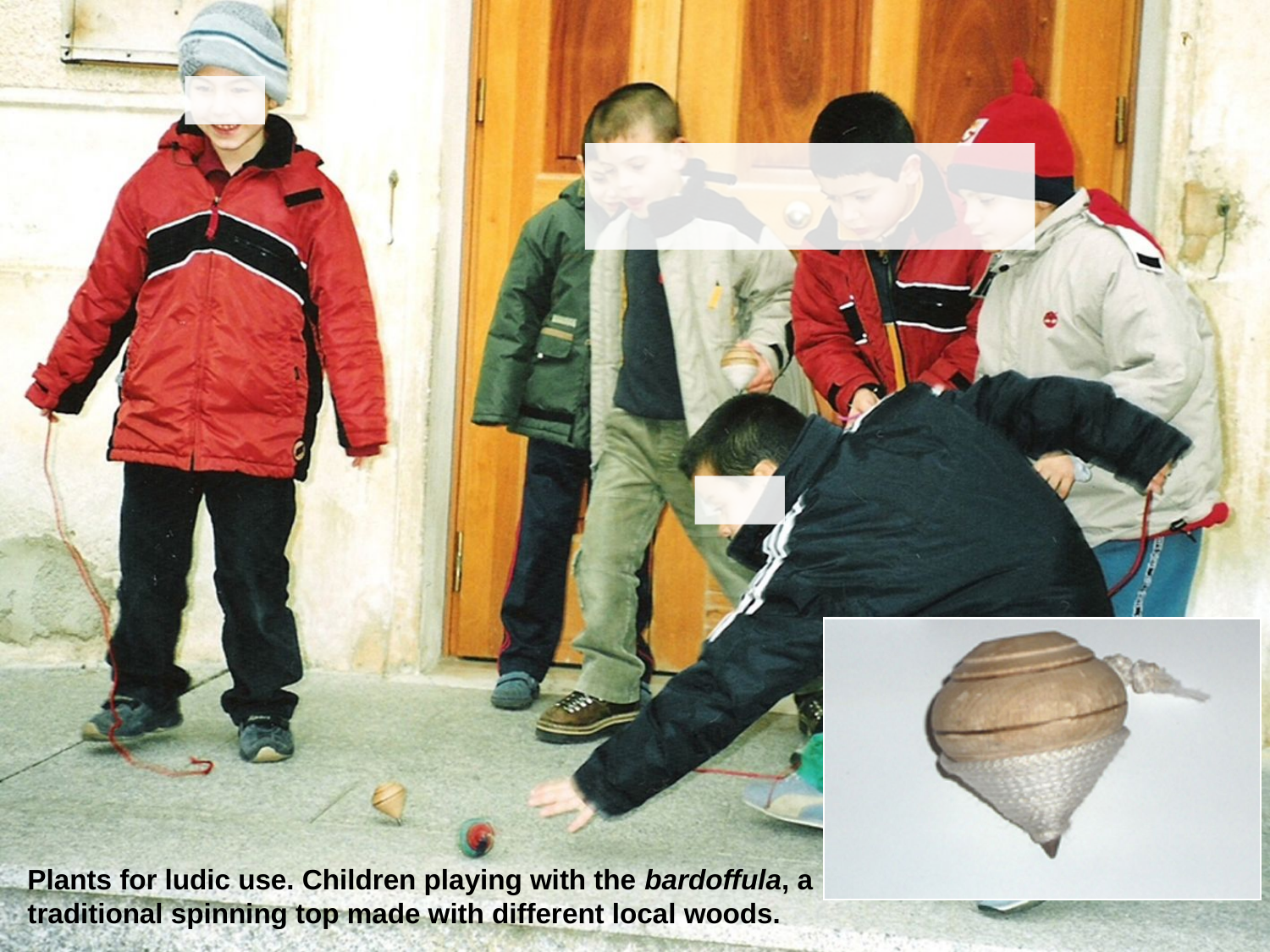

Plants for ludic use. Children playing with the bardoffula, a traditional spinning top made with different local woods.

## Slide 4
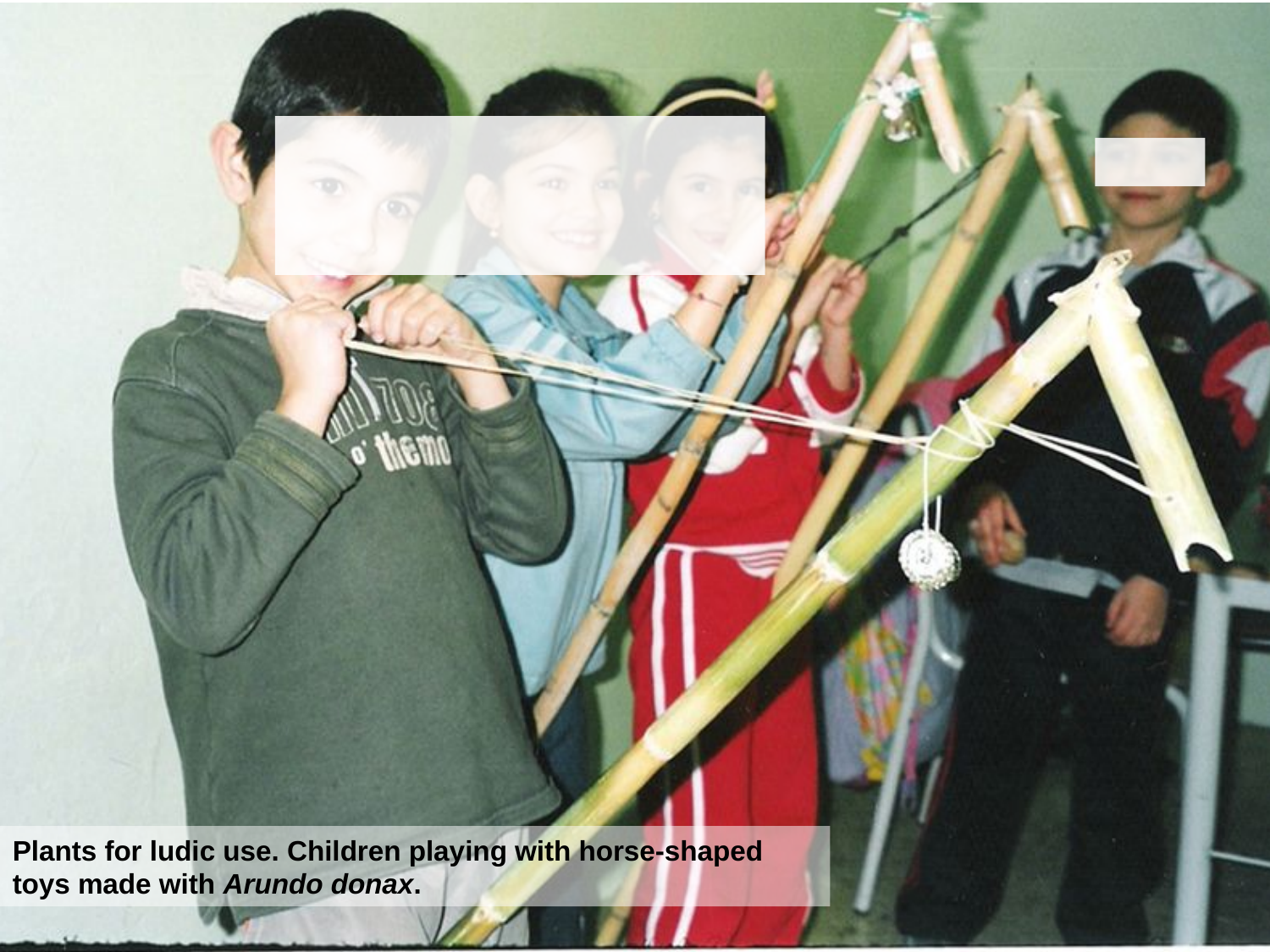

Plants for ludic use. Children playing with horse-shaped toys made with Arundo donax.

## Slide 5
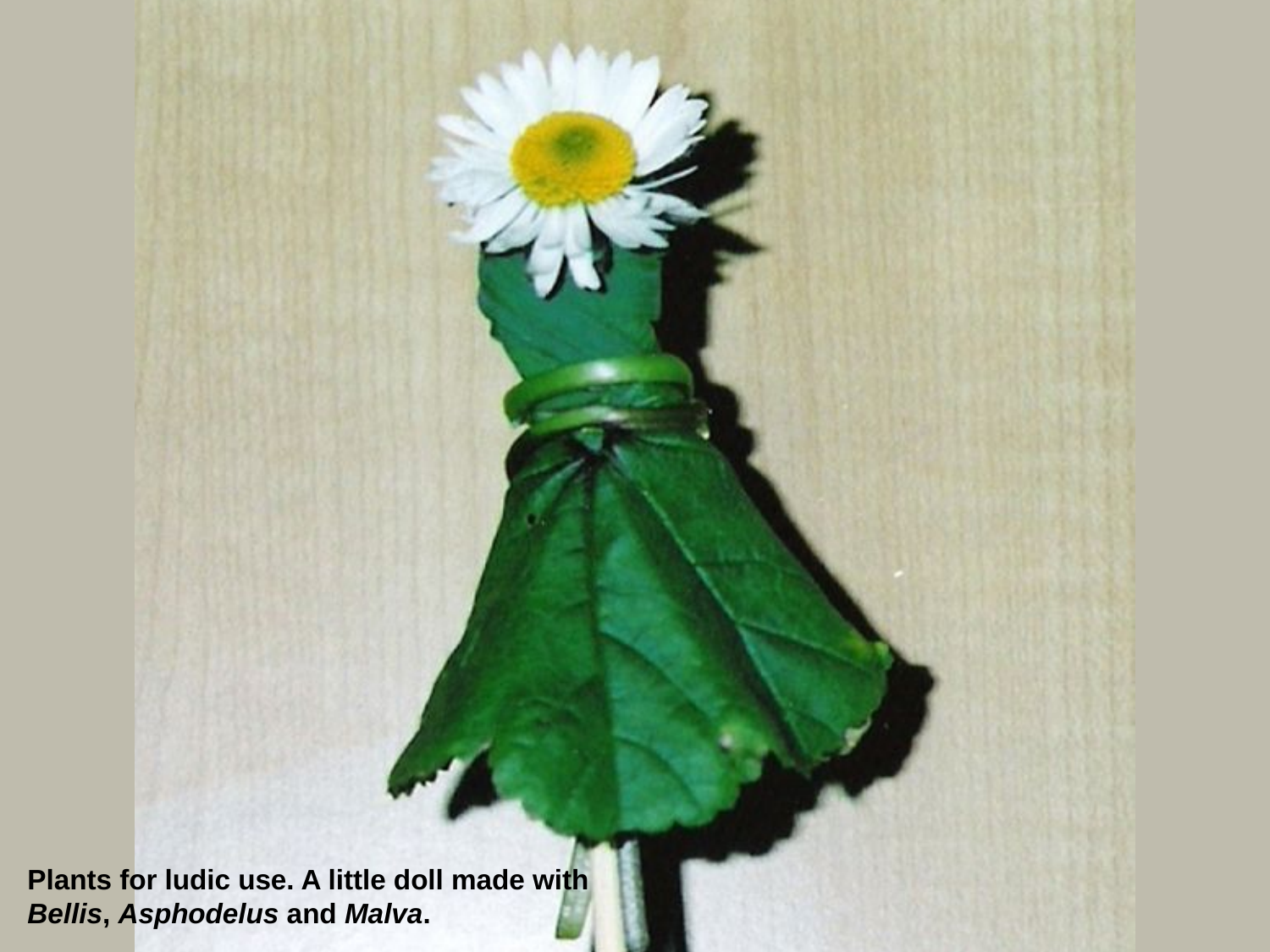

Plants for ludic use. A little doll made with Bellis, Asphodelus and Malva.

## Slide 6
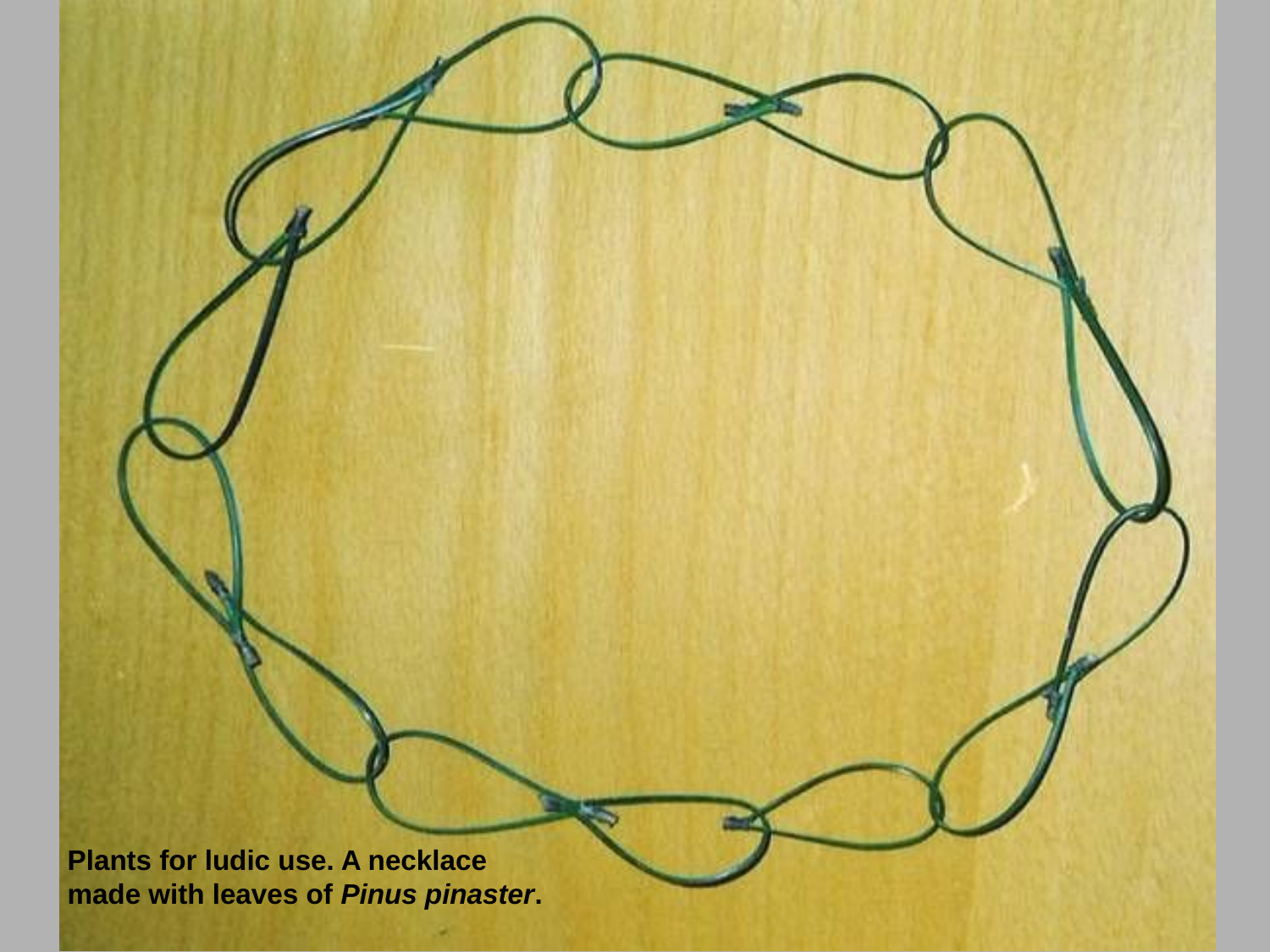

Plants for ludic use. A necklace made with leaves of Pinus pinaster.

## Slide 7
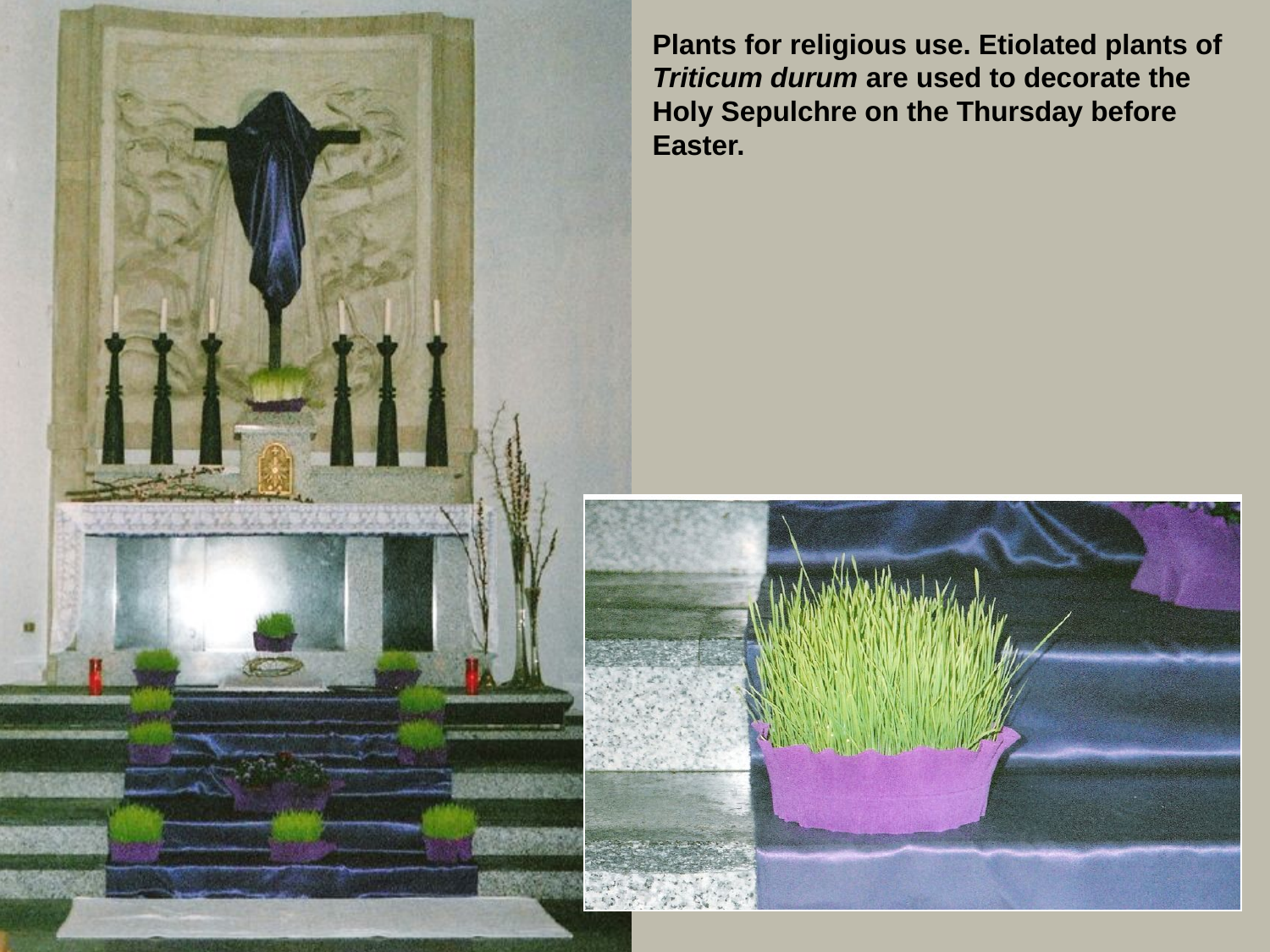

Plants for religious use. Etiolated plants of Triticum durum are used to decorate the Holy Sepulchre on the Thursday before Easter.

## Slide 8
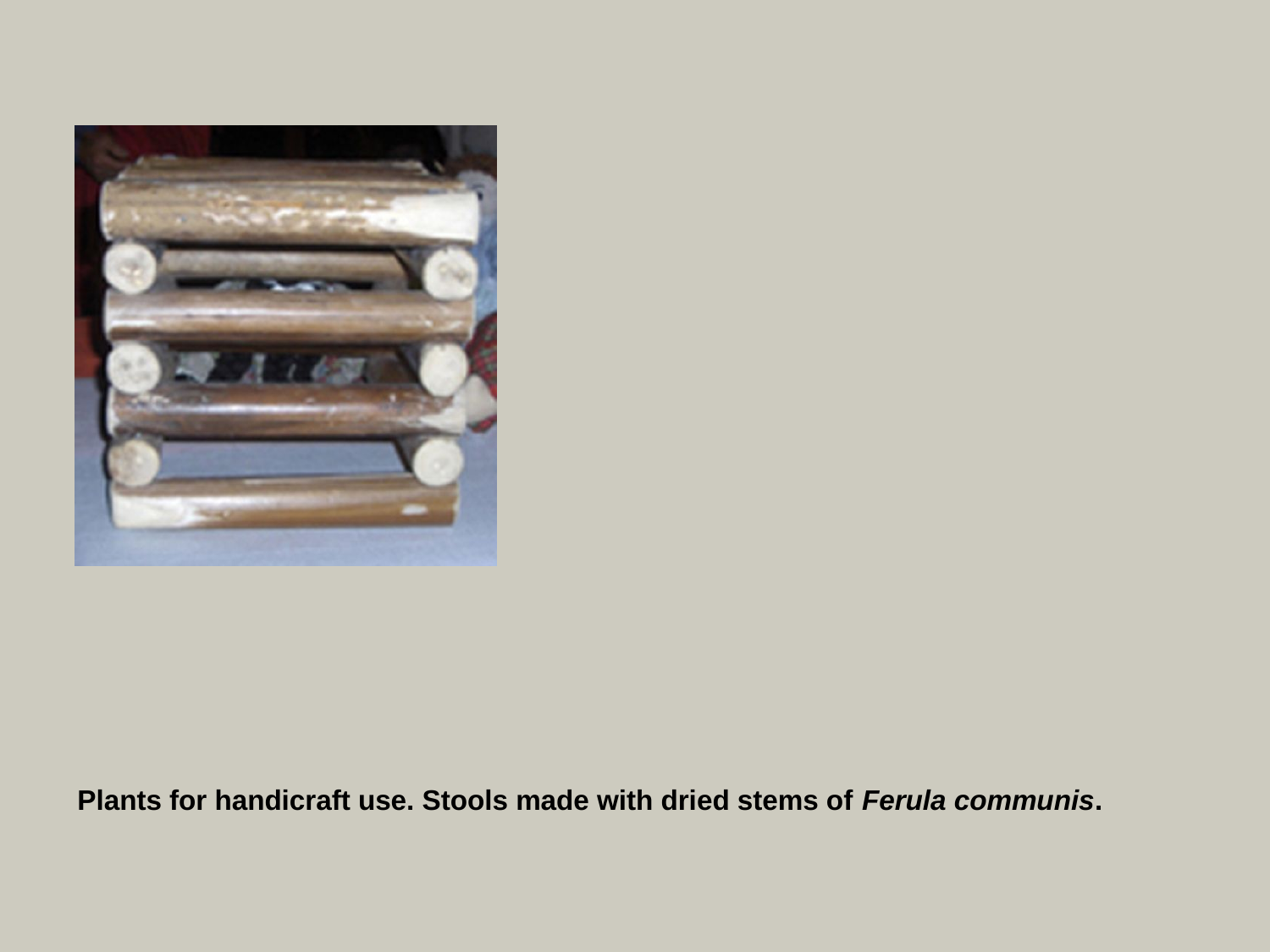

Plants for handicraft use. Stools made with dried stems of Ferula communis.
